# Therapy of solid tumors using probiotic Symbioflor-2 – restraints and potential

**DOI:** 10.18632/oncotarget.8027

**Published:** 2016-03-10

**Authors:** Dino Kocijancic, Sebastian Felgner, Michael Frahm, Ronja-Melinda Komoll, Aida Iljazovic, Vinay Pawar, Manfred Rohde, Ulrike Heise, Kurt Zimmermann, Florian Gunzer, Juliane Hammer, Katja Crull, Sara Leschner, Siegfried Weiss

**Affiliations:** ^1^ Department of Molecular Immunology, Helmholtz Centre for Infection Research, Braunschweig, Germany; ^2^ Central Facility for Microscopy, Helmholtz Centre for Infection Research, Braunschweig, Germany; ^3^ Mouse-Pathology Service Unit, Helmholtz Centre for Infection Research, Braunschweig, Germany; ^4^ Symbio Gruppe GmbH & Co KG, Herborn, Germany; ^5^ Institute of Medical Microbiology and Hygiene, Dresden University of Technology, Dresden, Germany; ^6^ Institute of Immunology, Medical School Hannover, Hannover, Germany

**Keywords:** probiotic, Escherichia coli, bacteria mediated tumor therapy, cancer immune therapy, murine tumor model

## Abstract

To date, virulent bacteria remain the basis of most bacteria mediated cancer therapies. For clinical application attenuation is required. However, this might result in a drastically lowered therapeutic capacity. Herein we argue that the *E. coli* probiotic Symbioflor-2, with a history of safe application may constitute a viable tumor therapeutic candidate. We demonstrate that Symbioflor-2 displays a highly specific tumor targeting ability as determined in murine CT26 and RenCa tumor models. The excellent specificity was ascribed to reduced levels of adverse colonization. A high safety standard was demonstrated in WT and Rag1^−/−^ mice. Thus, Symbioflor-2 may represent an ideal tumor targeting delivery system for therapeutic molecules. Moreover, Symbioflor-2 was capable of inducing CT26 tumor clearance as result of an adjuvant effect on tumor specific CD8^+^ T cells analogous to the *Salmonella* variant SL7207. However, lower therapeutic efficacy against RenCa tumors suggested a generally reduced therapeutic potency for probiotics. Interestingly, concurrent depletion of Gr-1^+^ or Ly6G^+^ cells installed therapeutic efficacy equal to SL7207, thus highlighting the role of innate effector cells in restraining the anti-tumor effects of Symbioflor-2. Collectively, our findings argue for a strategy of safe strain application and a more sustainable use of bacteria as a delivery system for therapeutic molecules.

## INTRODUCTION

Treating tumors with infectious agents exemplifies a longstanding strategy [1–3]. It was recognized and deployed on account of a mere beneficial correlation between bacterial infection and tumor regression [4–7]. Therapeutic efficiency with coincidental or intentional use of bacteria like *Streptococcus pyogenes* and *Clostridium perfringens* suggested virulent bacteria as mediators for tumor therapy. However, lack of controllability rendered such treatments a gamble of life and death. Consequently, early efforts resolved to heat killing of the bacteria and local application, as documented in the pioneering work of William B. Coley in the early twentieth century [8, 9]. However, such a strategy would leave out the therapeutic advantage of tumor colonization affiliated with systemic application, and shown for a wide array of bacterial species including *Salmonella spp., Clostridium spp., Bifidobacterium spp., Corynebacterium spp.* and *Escherichia spp.* [10, 11].

Nowadays, mechanistic insight and modern techniques allow for installment of control and safety to bacteria in more sophisticated ways [12–14]. The possibility to genetically alter bacteria by attenuation and recombinant strengthening, allows for the tailoring of bacteria according to the needs of efficacy and safety [14–21]. Nevertheless, research efforts still struggle to reconcile both elements in the very same bacterial strain [22–25].

The gram-negative facultative anaerobic bacterium *Salmonella enterica* serovar Typhimurium has essentially prevailed as a therapeutic candidate since the millennium and remains subject to most investigations nowadays [26–30]. Intrinsically virulent, an absolute requisite for its application is attenuation. However, clinical studies with VNP20009, a highly attenuated variant of *Salmonella,* has failed upon systemic or even local application in cancer patients [31, 32]. Neither therapeutic efficacy nor tumor colonization could be achieved at the maximum tolerated dose upon systemic application. These studies demonstrate the fundamental problem of intrinsic virulence, as the particular safety measures may have prohibited therapeutic efficacy. Reconciling therapeutic effectiveness and safety of application within the same bacterial strain indeed remains a major challenge. One solution might be to reconsider which bacterial strains to deploy for bacteria mediated tumor therapy (BMTT). Ensuring intrinsic bacterial safety would be an appropriate starting point as genetic engineering might subsequently allow for recombinant installation of therapeutic effects [33–35]. Thus, readjusting focus from virulent to probiotic bacteria might be a more rational strategy, provided, that some of the intrinsic therapeutic properties observed for virulent strains are preserved.

*E. coli* is a bacterial species of *Enterobacteriaceae* closely related to *Salmonella*. This provides high transferability of knowledge and material. Accordingly, several probiotic strains of *E. coli* have recently been investigated for BMTT. They demonstrated a preserved ability to colonize tumor tissue with great specificity and few side effects [36, 37]. However, aside from the colonizing aspect, intrinsic therapeutic effects have not been elaborately described thus far.

*E. coli* Nissle (EcN; commercially available as Mutaflor), the most widely explored *E. coli* probiotic for this and other therapeutic applications is known to contain virulence factors that could potentially render this strain unsuitable for systemic application in BMTT [38–42]. Therefore, one should explore other probiotic *E. coli* representatives like Symbioflor-2 (DSM17252). Since its isolation in 1956, Symbioflor-2 has a documented history of safe oral application, and was allocated to risk group 1 by the German authorities in 2011 [43, 44]. Symbioflor-2 comprises six genotypically similar *E. coli* strains denominated: G1/2, G3/10, G4/9, G5, G6/7, and G8 [45]. Thus far, the potential of Symbioflor-2 strains in BMTT remained uncharacterized.

In this study, we explore the potential of Symbioflor-2 strains for BMTT using a murine transplantable tumor model. With this model, our group has extensively described the ability of the *Salmonella* variant SL7207 to induce complete tumor regression of CT26 tumors and to establish immunological memory against the tumor [46–51]. SL7207 displays tumor colonization with a specificity of >1000:1 compared to healthy organs like liver and spleen. Furthermore, for mechanistic explanations regarding tumor invasion, colonization, and anti-tumor response, SL7207 is a fair representative of vastly exploited attenuated *Salmonella* variants applied for BMTT including A1-R, LVR01, χ4550, *ΔppGpp*, YB-1, and VNP20009 [23, 29, 52–57]. Altogether, it represents a solid standard for evaluating probiotic alternatives like Symbioflor-2.

We investigated Symbioflor-2 regarding therapeutic safety upon systemic application and therapeutic efficacy in the immunogenic CT26 and more resilient RenCa murine tumor models. Moreover, we explored its susceptibility to innate defense mechanisms. Overall the study reveals that Symbioflor-2 provides a safer alternative to *S.* Typhimurium, and even to EcN, in BMTT. Thus Symbioflor-2 offers a basis as bacterial bio-vehicle for delivery of therapeutic molecules.

## RESULTS

### Colonization of CT26 tumors by *E. coli* probiotics

**Figure 1:**
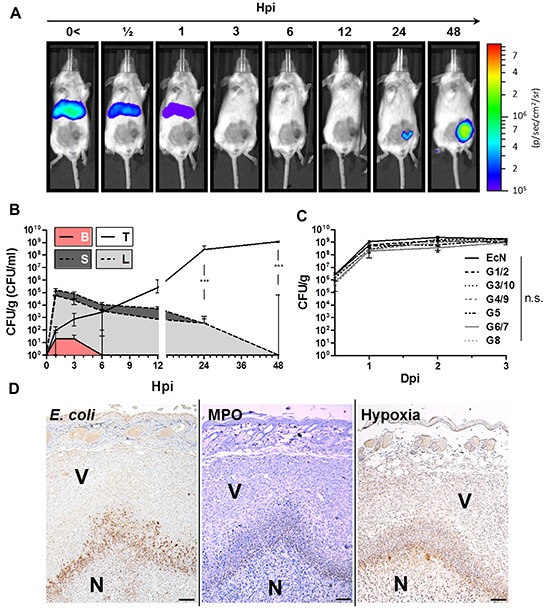
Early colonization profile of Symbioflor-2 in a murine CT26 tumor model 5×10^5^ CT26 tumor cells were inoculated s.c. into wild-type mice. After 10 days, the mice were infected with 5×10^6^ probiotic *E. coli*. **A.** Distribution kinetic of strain G1/2 in a murine host upon systemic inoculation. G1/2 contains the plasmid pHL304 encoding the *luxCDABE* operon, and was detected using an *in vivo* imaging system (IVIS). **B.** Colonization profile displaying CFU counts in blood (B), liver (L), spleen (S), and CT26 tumor (T) upon systemic inoculation of G1/2 pHL304. **C.** Comparison of CT26 tumor colonization between individual Symbioflor-2 strains and EcN on selected time points. **D.** IHC staining of consecutive CT26 tumor cross sections 48 hpi with pooled Symbioflor-2 strains reveals a pattern of colocalization between *E. coli*, hypoxic regions and neutrophil accumulation. Specific antibodies were used to detect *E. coli*. Neutrophilic granulocytes were identified via myeloperoxidase (MPO) staining and regions of hypoxia were stained for presence of pimonidazole metabolites. V and N denote viable and necrotic tumor tissue, respectively. Dpi/ hpi denote days/ hours post infection. Representative IVIS and IHC images are displayed. Scale bar corresponds to 100 μm. N=3-5. Median ± range.

Many related and unrelated bacterial species, including various strains of *Salmonella* and *E. coli* have been demonstrated to colonize tumor tissue upon systemic infection [10]. We investigated if this ability would also be exhibited by Symbioflor-2. Subcutaneous implantation of CT26 colon carcinoma cells to BALB/c mice was used as a tumor model to study their colonizing ability and therapeutic effects. In a first instance, we assessed the *in vivo* distribution of strain G1/2, a representative of Symbioflor-2, in tumor bearing mice upon i.v. inoculation with a dose of 5×10^6^. Comparable doses had previously been deployed for SL7207 in similar studies [47, 49]. For noninvasive live detection, a reporter plasmid conferring constitutive Lux expression was transformed into the G1/2 strain. Using this setup, a bacterial signal was detected almost immediately upon infection in the upper quadrants of the murine host (in a coronal plane) signifying colonized spleen and liver. The signal steadily decreased until vastly undetectable by 3 hpi (Figure 1A). By 24 hpi, the signal reappeared, now within the transplanted CT26 tumor which further intensified at 48 hpi (Figure 1A). Importantly, transformants were stable *in vivo* as assessed by selective plating, thus validating the correlation between Lux intensity and bacterial localization (Supplementary Figure S1).

As *in vivo* imaging may hold bias, we confirmed our colonization profile by standard quantitative plating. The early kinetic profile revealed that the majority of the G1/2 inoculum was allocated to accessory organs like liver and spleen reaching CFU counts of 1×10^5^ per g tissue by 1 hpi. In blood, low counts of 10 CFU/ml were detected at 1 and 3 hpi, until completely cleared by 6 hpi (Figure 1B). Interestingly, and supporting the IVIS results, CFU counts in liver and spleen steadily decreased below detection within 48 hpi (Figure 1B). Thus, bacteria are quickly cleared from these systemic target locations [58]. Conversely, initial counts of 10 CFU/g in the CT26 tumor tissue by 1 hpi exponentially increased over 48 hours ultimately reaching a stable plateau of 1×10^9^ CFU/g tumor (Figure 1B, 1C). Similarly efficient and consistent tumor colonization was observed with all Symbioflor-2 constituent strains as well with Mutaflor (EcN) (Figure 1C). Moreover, rapid clearance and retraction from liver and spleen proved to be generic to all *E. coli* probiotics tested in this study (Supplementary Figure S2). The consistent colonization profile displayed by all Symbioflor-2 constituents, along with their reported close phylogeny [45, 59], allowed us to proceed with pooled Symbioflor-2 in further investigations.

In the tumor, *Salmonella* SL7207 has been described to reside close to a central necrotic core most likely dictated by growth preference in a hypoxic/ anoxic niche [48, 49, 60]. Specific staining for *E. coli* in histological sections of CT26 tumors isolated 48 hpi showed a similar profile. The probiotics were found to reside adjacent to, and within, the central necrosis. They co-localized with hypoxic regions as determined by specific staining for pimonidazole metabolites on a consecutive section (Figure 1D). In line with earlier reports, a necrotic niche in the tumor substantiated upon systemic infection (Supplementary Figure S3). Furthermore, Westphal et al. reported that neutrophils play a pivotal role in bacterial containment and localize around the bacteria in the interphase between necrotic and viable regions of the tumor [48]. Analogously, a high density of myeloperoxidase positive neutrophils overlapping with the bacteria could be identified at a similar location (Figure 1D, Supplementary S3). Altogether, the results suggest that characteristics regarding SL7207 tumor colonization can be applied to Symbioflor-2 as well.

### Colonization of RenCa tumors by *E. coli* probiotics

**Figure 2:**
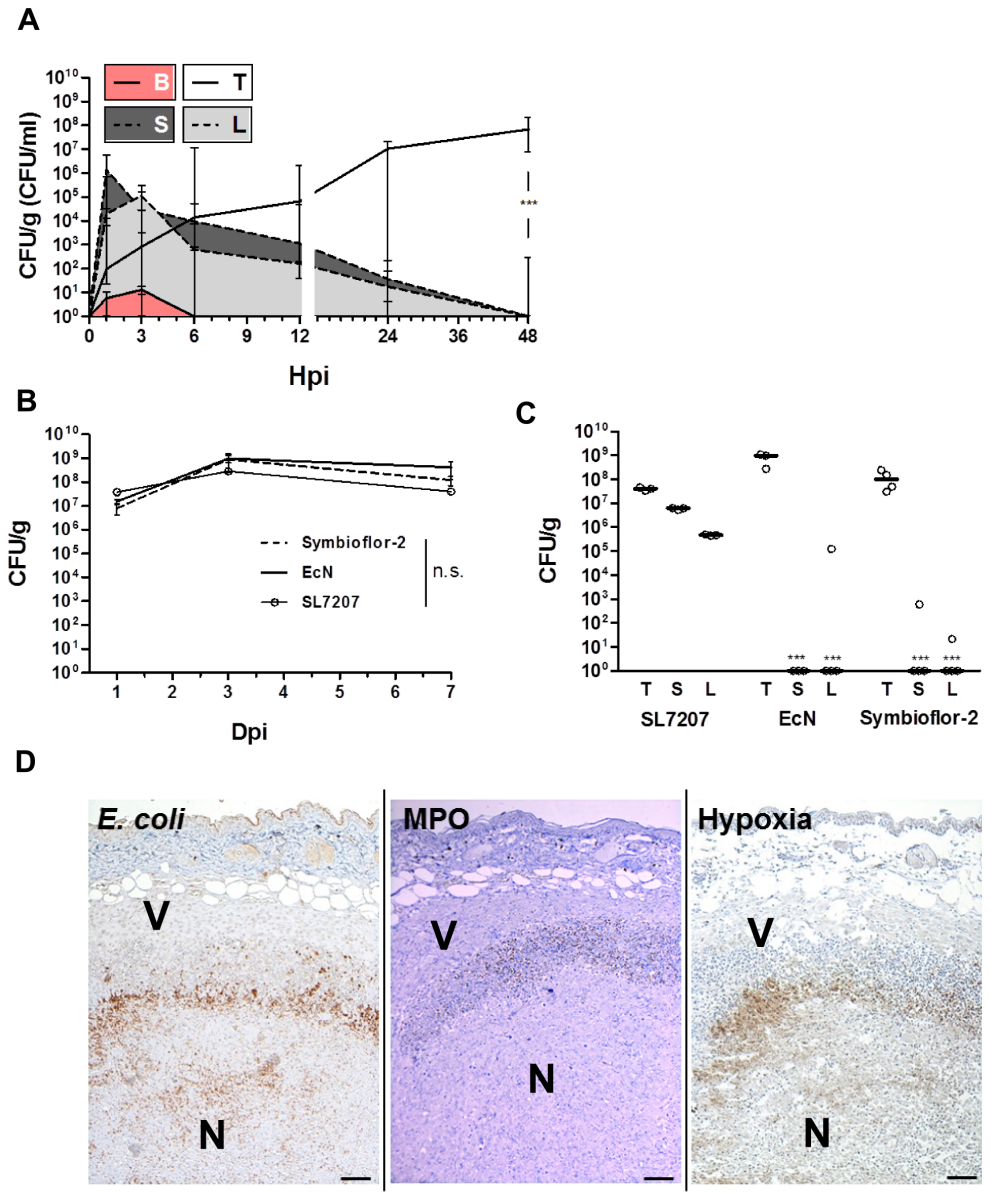
Validating colonization by Symbioflor-2 in a RenCa tumor model demonstrates persistence of colonization and intrinsic tumor specificity following systemic inoculation Wild-type mice received a subcutaneous injection of 1.5×10^6^ RenCa tumor cells. “Symbioflor-2” constitutes strains G1/2, G3/10, G4/9, G5, G6/7, and G8 pooled at equal ratios. **A.** Colonization profile displaying CFU counts in blood (B), liver (L), spleen (S), and RenCa tumor (T) upon systemic inoculation of strain G1/2. **B.** Comparison of RenCa tumor colonization between Symbioflor-2, EcN, and SL7207 over 7 days of infection. **C.** Specificity of RenCa tumor colonization 7 dpi between Symbioflor-2, EcN and SL7207. **D.** IHC staining of consecutive RenCa tumor cross sections 48 hpi with Symbioflor-2. *E. coli* were stained using a specific antibody, MPO activity was indicative of neutrophil activity and regions of hypoxia were stained for presence of pimonidazole metabolites. V and N denote viable and necrotic regions of the tumor, respectively. Representative images are displayed. Scale bar corresponds to 100 μm. N=3-5. Median ± range.

To validate the finding that Symbioflor-2 exhibits an intrinsic ability of tumor targeting and colonization, and to directly evaluate its efficiency and specificity relative to SL7207, we applied another less exploited and more rigid syngeneic carcinoma model, RenCa. The Symbioflor-2 representative strain G1/2 maintained an early colonization profile similar to that seen before with CT26 and a transient colonization of normal tissues (Figure 2A). CFU counts in the tumor increased steadily within 48 hpi while colonization of spleen and liver disappeared analogous to prior observations (Figure 2A).

RenCa tumor colonization by *E. coli* probiotics Symbioflor-2 (pooled strains) and EcN was equally efficient as SL7207, and persisted over 7 dpi (Figure 2B). Considering adverse colonization, at 7 dpi SL7207 had established chronic high CFU counts above 1×10^5^ in liver and spleen, while Symbioflor-2 and EcN CFUs were significantly reduced, and for a great majority of animals cleared altogether (Figure 2C).

The ratio of CFU counts in the tumor relative to CFU's in normal tissue represents a measure of specificity frequently used. By this standard SL7207 was shown to colonize RenCa tumors with a specificity of 10:1 or 100:1 relative to spleen and liver, respectively. Ratios higher than 10^8^:1 were obtained for Symbioflor-2 and EcN, thus exhibiting an even greater specificity for this tumor (Figure 2C).

Histological analyses of infected RenCa tumors revealed a localization profile similar to that demonstrated for CT26 (Figure 2D, Supplementary Figure S3). Taken together, the observations made with the CT26 tumor applied to the RenCa tumor as well. This confirms the general tumor targeting ability of Symbioflor-2. As tumor colonization by SL7207 and the probiotics was achieved with equal efficiency and with similar localization, it also demonstrates that pathogenicity per se does not constitute a prerequisite for tumor invasion and colonization.

### *E. coli* probiotics exhibit an improved safety profile upon systemic infection

**Figure 3:**
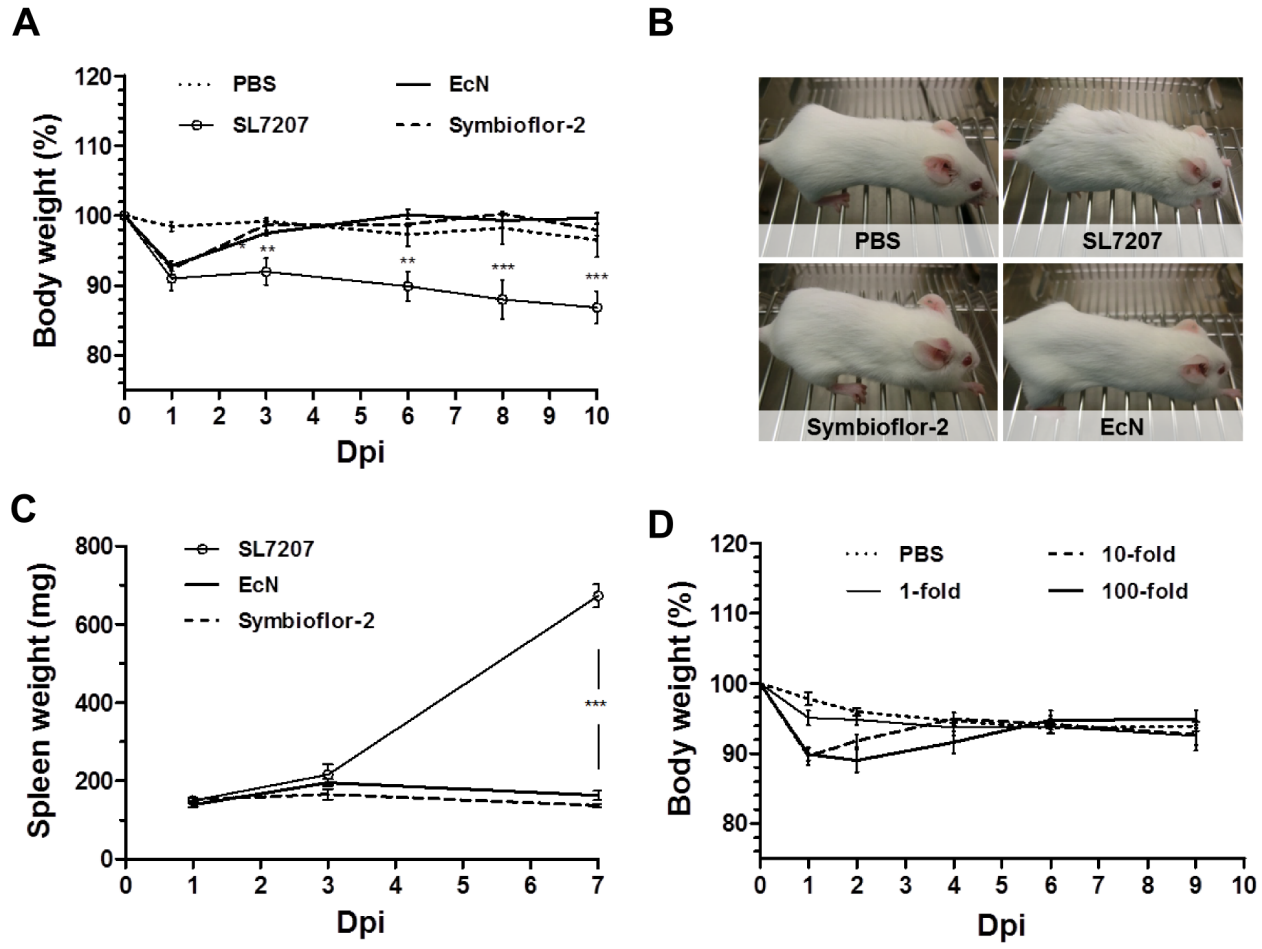
Transient manifestation of infection in the murine host following systemic inoculation with *E. coli* probiotics The host phenotype was evaluated in tumor bearing mice in response to infection with Symbioflor-2, EcN or SL7207. **A.** Development of host body weight over the course of systemic infection. **B.** Phenotypic appearance of wild-type mice upon infection. SL7207 infection causes fatigue and a disrupted fur coat, while probiotic infection does not cause observable manifestations. Representative images from 3 dpi are displayed. **C.** Progression in spleen weight of infected mice. **D.** Dose-response in host body weight with Symbioflor-2 infection. 1-, 10-, and 100- fold inoculum signify an infection dose of 5×10^6^, 5×10^7^ and 5×10^8^, respectively. N= 5-8. Mean ± SEM.

Safety of administration is requisite for systemic application of bacteria in a therapeutic context. This has proven an obstacle in preclinical models and an impediment for delivery in clinical trials using attenuated *Salmonella* [31, 32]. One may hypothesize that probiotics are more likely to preserve a safe phenotype in this artificial mode of infection bypassing the intestinal barrier. The earlier observations that *E. coli* probiotics, in contrast to SL7207, accumulate only transiently in liver and spleen is a first indication of an improved safety phenotype of such bacteria (Figure 1B, 2A, Supplementary Figure S2). To gather more details, we evaluated the host phenotype over the course of infection. Systemic infection with SL7207 or probiotics was equally reflected in the body weight, with an initial loss of <10% by day 1 p.i. (Figure 3A). While progressing during salmonellosis, the weight loss during infection with Symbioflor-2 or EcN proved to be transient. Regaining of weight led to normalization upon 3 dpi (Figure 3A).

When evaluating the general appearance and behavior of the animals, infection with probiotics resulted in a healthy host phenotype which stands in great contrast to the manifestations of salmonellosis. Here, a disrupted fur coat and overall fatigue was observed (Figure 3B). These manifestations are likely ascribed to a variety of pathological changes, some of which may result from the colonization of healthy organs. For example, a state of hypertrophy (splenomegaly) correlated with the presence of SL7207 in the spleen. Conversely, the absence of colonization of these organs by probiotics prevented such manifestations (Figure 3C).

Therapeutic effects in a prospective clinical situation might require an increased inoculum. Thus, tolerance to high infection doses may prove advantageous. Administering 10- and 100-fold of the original infection dose of Symbioflor-2 was well tolerated by the murine host. A similar body weight profile dictated by recovery was sustained from either dose. Only the period of transient weight loss was prolonged (Figure 3D). Moreover, the host did not display apparent signs of morbidity, but retained a phenotype similar to a lower dose infection (data not shown). In addition, although CFU counts in blood, liver and spleen were initially significantly increased upon infection with a 100-fold infection dose, they retained a profile of clearance from these locations (Supplementary Figure S4). Collectively, Symbioflor-2 was tolerated well by the murine host upon systemic infection. No apparent detrimental signs of morbidity were observed at any point. This high safety standard would allow implementation of a systemic mode of inoculation that could improve therapeutic application via targeting of multiple and inaccessible tumors from a distant inoculation site.

### Symbioflor-2 is unable to form biofilms *in vivo* and is susceptible to innate defense mechanisms

**Figure 4:**
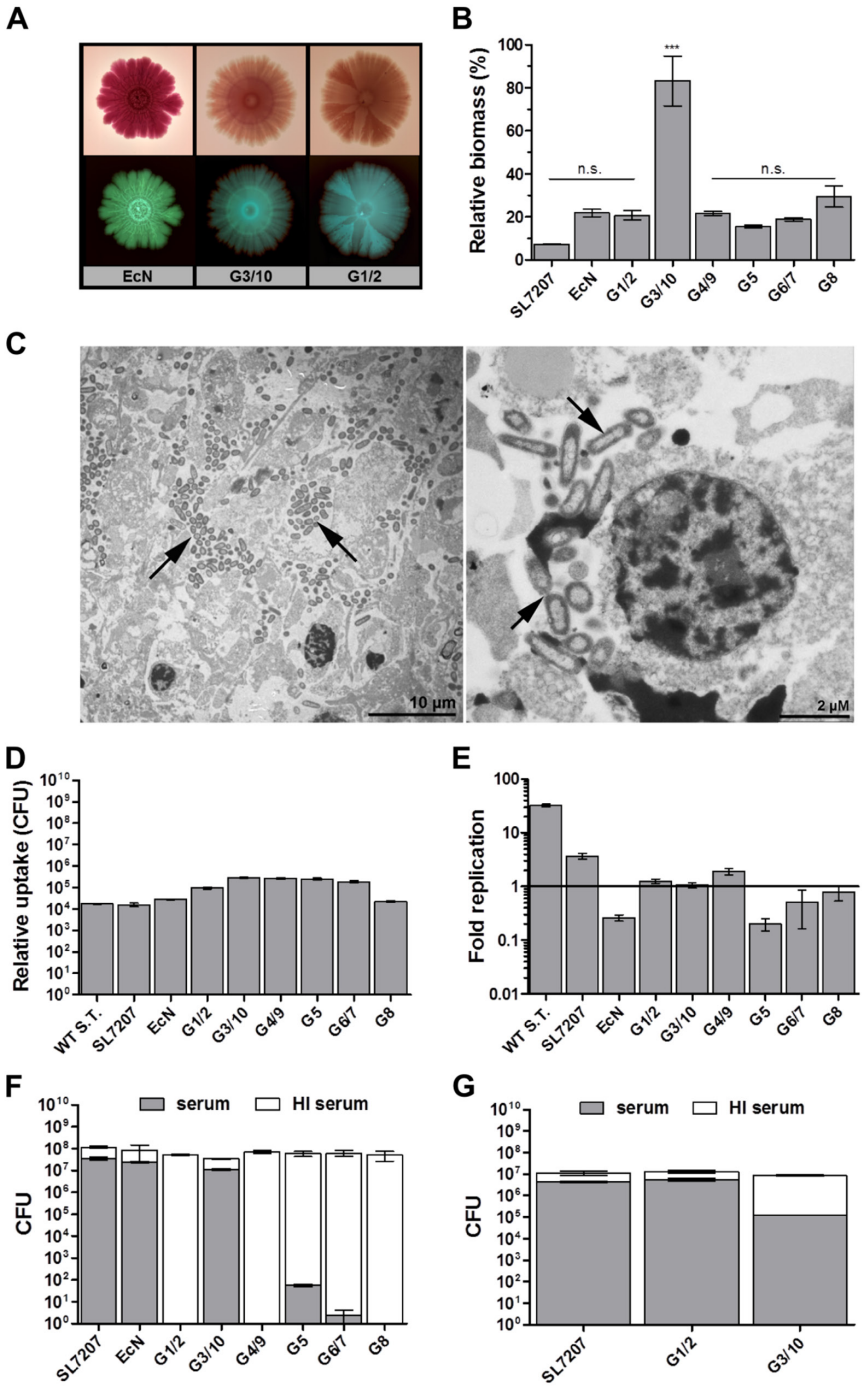
Symbioflor-2 fitness parameters; biofilm forming capacity, complement sensitivity, phagocytosis and intracellular survival **A.** *In vitro* biofilm forming capacity of representative Symbioflor-2 strains and EcN developed over 20 days on spiked agar plates. Bottom and top rows represent positively and negatively scanned biofilms, respectively. **B.** Quantification of static biofilm formation by Symbioflor-2 strains, EcN and SL7207 relative to a biofilm-positive *Pseudomonas aeruginosa* control strain PA14 (100%), as determined through crystal violet staining of a 48 h culture deposit. N=8. **C.** Ultrathin sections of a Symbioflor-2 colonized CT26 tumor section isolated 48 hpi. Arrow heads point to the colonizing bacteria. Representative images are displayed. **D.** Bacterial uptake by *ex vivo* matured BMDMs, as determined through relative comparison of intracellular bacteria 1 hpi in a co-culture gentamicin survival assay. **E.** Intracellular survival/ replication of the bacteria in BMDMs as determined through intracellular CFU counts 18 hpi and expressed relative to levels of uptake 1 hpi. N=5. Wild-type *Salmonella* strain ATCC14028 (WT S.T.) was included as a positive control. **F.** Bacterial susceptibility to lysis by human complement. Treatment with heat inactivated (HI) human complement constitutes a control for viable bacteria and relative inocula. **G.** Sensitivity to canine complement lysis for G1/2, G3/10 and EcN. N=5. Mean ± SEM and median ± range were deployed on linear and log scales, respectively.

We asked if functional analyses may support this well tolerated bacterial phenotype. An ability to form biofilm has been shown to confer resistance to anti-microbial interventions such as protection from innate defense mechanisms or antibiotic treatment [51, 61–63]. Aside from a preserved capacity to form biofilm on a plate over 20 days (Figure 4A, Supplementary Figure S5), the *E. coli* probiotics were all, with the exception of strain G3/10, unable to deposit extracellular matrix material or extracellular polymeric substances (EPS) *in vitro* in a standard quantitative biofilm assay (Figure 4B). A similar phenotype was observed *in vivo,* as no apparent EPS could be identified in the tumor tissue via transmission electron microscopy with Symbioflor-2 (Figure 4C). Rather, single bacteria were found to reside in necrotic regions, presumably scavenging cellular debris (Figure 4C). This phenotype was also observed upon single strain infection using individual Symbioflor-2 strains (data not shown). Taken together, *E. coli* probiotics did not display a biofilm phenotype *in vivo*, and should thus be well susceptible to antibiotic control [64].

To address the susceptibility to innate effector mechanisms, we first evaluated the performance of Symbioflor-2 strains regarding phagocytic uptake. Phagocytic uptake was assessed using primary BMDMs and the immortalized macrophage-like cell line J774. Similar results were obtained for both types of cells. Phagocytic uptake was comparable between all *E. coli* probiotic and *Salmonella* strains (Figure 4D, Supplementary Figure S6). Moreover, all *E. coli* probiotics displayed intracellular survival. In contrast to wild type *Salmonella* or SL7207, only marginal intracellular replication was observed for the probiotics (Figure 4E, Supplementary Figure S6). Thus, they should be considered controllable by macrophage uptake *in vivo*.

We extended our analyses to complement sensitivity. The complement system is an immune component effective in combating foreign circulatory pathogens. As murine complement has been deemed experimentally difficult to handle in experimental assays [65–67], we evaluated the resistance of our strains towards lysis by human and canine complement. Surprisingly, the majority of Symbioflor-2 strains displayed a deviating phenotype compared to EcN and SL7207. They exhibited sensitivity to lysis by human complement (Figure 4F). Again, G3/10 proved an exception, as it was resistant to human complement (Figure 4F). However, a contrasting result was achieved when testing sensitivity to lysis by canine complement. Here, only Symbioflor-2 strain G1/2 turned out to be resistant (Figure 4G), hence demonstrating that complement sensitivity is host-specific. Sensitivity to complement may prove a potential obstacle for systemic delivery in a human host during transit in the circulation. Therefore the relative importance of this phenotypic quality will need to be evaluated further. For the moment however, Symbioflor-2 contains strains of either phenotype.

### Differential pathogenicity of *E. coli* probiotics in immune compromised hosts

**Figure 5:**
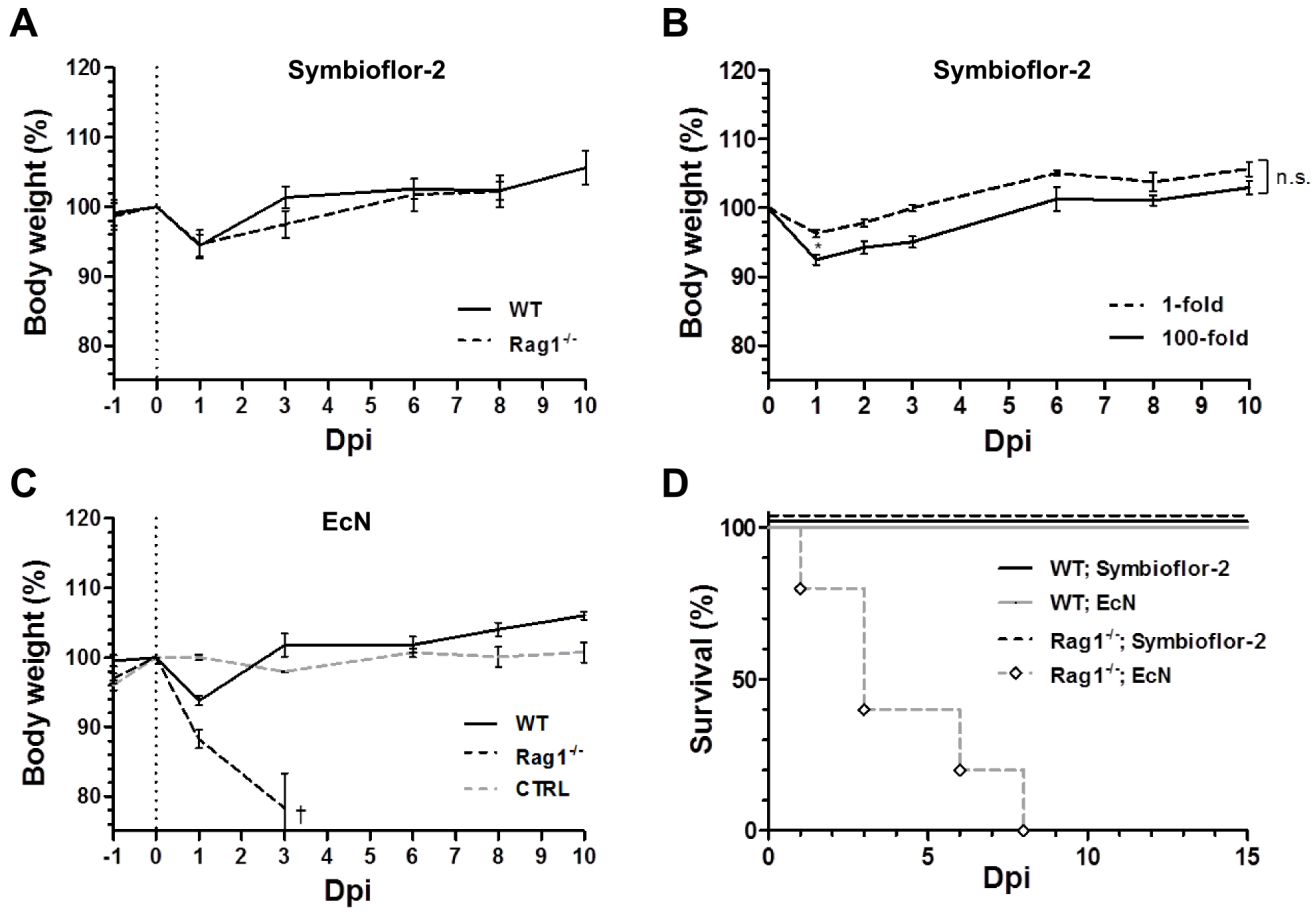
Rag1^−/−^ mice succumb to infection by EcN, while tolerable to Symbioflor-2 Rag1^−/−^ mice were infected i.v. with 5×10^6^ Symbioflor-2 or EcN upon which the host phenotype was evaluated. **A.** Host body weight comparison between Wild-type (WT) and Rag1^−/−^ mice over the course of systemic infection with Symbioflor-2. **B.** Tolerability to high dose (100-fold) Symbioflor-2 infection in Rag1^−/−^ mice. **C.** WT and Rag1^−/−^ host body weight profile upon systemic inoculation with EcN. Rag1^−/−^ mice were analogously treated with PBS as control (denoted “CTRL”). **D.** Survival rate of WT and Rag1^−/−^ mice with probiotic infection. N=5-8. Mean ± SEM.

Clinical application would involve treatment of cancer patients that are often immune compromised due to an advanced state of disease or as a result of conventional treatments [68, 69]. Therapeutic bacteria should be able to cope with these conditions by maintaining a safe profile. We extended our evaluation to Rag1^−/−^ mice deprived of a functional adaptive immune system, thus mimicking an immune compromised state of cancer patients. As determined by a comparable body weight profile, Symbioflor-2 was equally tolerated in Rag1^−/−^ mice as in wild-type mice at the standard dose (Figure 5A). Moreover, 100-fold the original infection dose caused a dose response in the body weight of Rag1^−/−^ mice similar to that previously observed in wild-type mice (Figure 5B, 3D). Regardless the dose, the Rag1^−/−^ mice remained discernably unaffected by the infection (data not shown). Altogether Symbioflor-2 infection was tolerated to an equal extent in Rag1^−/−^ mice as in wild-type BALB/c mice, thereby adding to its safety profile.

To our surprise EcN caused pathology in immune compromised Rag1^−/−^mice. Although experiments in wild type mice suggested a safe phenotype, the profile in Rag1^−/−^ mice revealed a dramatic weight loss with resultant mortality or a humane endpoint in the majority of mice by 3 dpi (Figure 5C, 5D). We wondered if this pathology might be caused by the known virulence factor Colibactin (*clb*) of EcN, a genotoxin thus far known to cause DNA damage and pre-stages of colorectal cancer (CRC) [40]. However, the mutant strain EcN *Δclb* exhibited the same phenotype as the parental strain (Supplementary Figure S7). This detrimental observation narrows the safety profile of EcN. The proneness of EcN to pathology necessitates further investigations before concluding on its therapeutic suitability.

### *E. coli* probiotics display a limited intrinsic efficacy in tumor therapy

**Figure 6:**
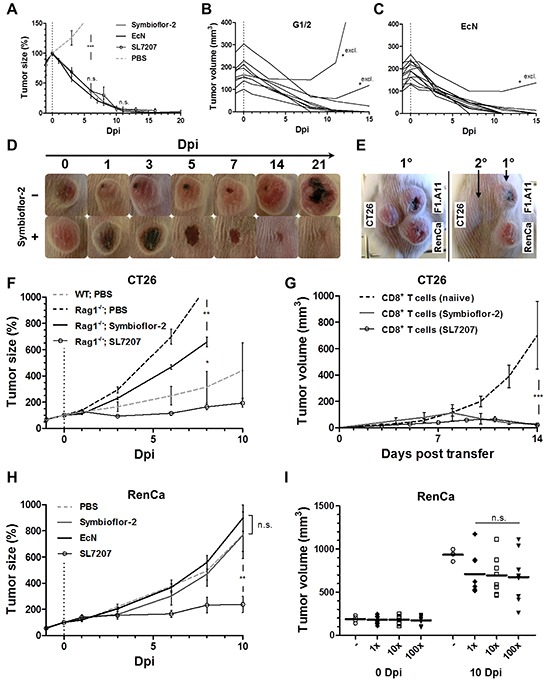
Therapeutic effects by *E. coli* probiotics include the induction of a tumor-specific CTL-response sufficient for CT26 tumor clearance, while inadequate for RenCa control Therapeutic effects against CT26 and RenCa were evaluated upon single-dose systemic bacterial infection using *E. coli* probiotics. **A.** Kinetic of CT26 tumor regression in WT mice following systemic infection with Symbioflor-2, EcN or SL7207. **B, C.** CT26 tumor development profile for individual replicates infected with Symbioflor-2 representative G1/2 or EcN. Replicates marked “*excl.” were excluded from the mean in (A). **D.** Macroscopic profile of a regressing CT26 tumor. Representative time points and cropped images are displayed. **E.** Re-challenge (2°) with the same tumor cell line (CT26) upon successful CT26 tumor clearance in response to probiotic infection. A course of antibiotic treatment ensured infection resolution prior to re-challenge three months later. Concurrent primary inoculations (1°) of syngeneic tumor cell lines RenCa and F1. A11 served as controls. Day 14 post tumor inoculation is displayed. **F.** CT26 tumor development in Rag1^−/−^ mice over the course of infection. **G.** CT26 tumor development in Rag1^−/−^ mice reconstituted with CD8^+^ T cells at the time of CT26 inoculation. 3×10^6^ CD8^+^ T cells were adoptively transferred from naiive or CT26 tumor bearing mice previously exposed to infection with Symbioflor-2 or SL7207, as indicated in the brackets. **H.** RenCa tumor development in WT mice over the course of infection with Symbioflor-2, EcN, and SL7207. **I.** Initial and end point tumor volume in WT mice deploying 1x, 10x, or 100x of the original infection dose of Symbioflor-2. N=6-8. Mean ± SEM.

As *E. coli* probiotics, and notably Symbioflor-2, have displayed a remarkable safety profile upon systemic infection in the murine host, we next asked if *E. coli* probiotics would retain the same therapeutic efficiency as reported for SL7207. We first compared the anti-tumor effect upon systemic infection in the CT26 model. Symbioflor-2 and EcN induced tumor regression with a kinetic similar to SL7207 (Figure 6A). However, in contrast to SL7207, probiotic infection exemplified by G1/2 and EcN gave rise to CT26 tumor regrowth in a small proportion of replicates, thereby suggesting a generally lower potency of these strains (Figure 6B, 6C). Macroscopically, anti-tumor effects by Symbioflor-2 were characterized by tumor darkening within 1 dpi, followed by necrosis formation, ulceration and tumor clearance by 14 dpi. Eventually, skin integrity was reinstalled over the subsequent weeks (Figure 6D). In support of a shared mode of action [49], *E. coli* probiotics induced an early, albeit lower TNFα response compared to SL7207 with subsequent tumor hemorrhage (Supplementary Figure S8).

As recently described, a specific anti-tumor immune response is stimulated by bacterial inoculation [70, 71]. Similarly, mice that had cleared the primary CT26 tumors after application of Symbioflor-2 did reject the same tumor cell line upon re-challenge (Figure 6E). This signifies that indeed a CT26-specific anti-tumor memory response had been induced by the infection. To confirm this causality, we evaluated the effect of Symbioflor-2 on CT26 tumor development in Rag1^−/−^ mice. Altogether, therapeutic effects were not as prominent as observed in wild-type BALB/c mice, i.e. at best only growth retardation was sustained (Figure 6F). Nevertheless, significant growth retardation in Rag1^−/−^ mice argues for presence of B/T cell independent effects which are triggered less effective by Symbioflor-2 compared to SL7207 (Figure 6F). Tumor colonization was comparable between Rag1^−/−^ and wild-type mice (Supplementary Figure S9).

It is by now well established that CD8^+^ T cells are the main effector cells governing an anti-tumor immune response [50]. To determine if this cell population was activated by the infection, we reconstituted Rag1^−/−^ mice with CD8^+^ T cells isolated from mice that had cleared CT26 tumors through infection and assessed CT26 tumor development. Tumor growth was initially retarded and completely abrogated around 7-10 days post adoptive transfer (Figure 6G). Interestingly, significant tumor retardation was also observed upon reconstitution with CD8^+^ T cells isolated from uninfected CT26 tumor bearing mice (Supplementary Figure S10B). This demonstrates the establishment of a tumor specific CD8^+^ T cell response pre infection, which is activated upon application of the bacteria. In contrast, reconstitution with CD4^+^ T cells from either source did not markedly affect tumor growth (Supplementary Figure S10C).

To further validate therapeutic efficacy, we next characterized RenCa tumor development upon systemic infection. Therapeutic effects were not as prominent as observed with CT26. Only SL7207 caused significant RenCa tumor retardation, while neither Symbioflor-2 nor EcN infection significantly affected tumor development (Figure 6H). This result was comparable between individual Symbioflor-2 strains (Supplementary Figure S11). Furthermore, a higher infection dose of Symbioflor-2 did not impact tumor development (Figure 6I). In extension, multiple re-infection also did not significantly improve therapeutic effects (Supplementary Figure S12A, S12B). However, reduction in the systemic TNFα response was imminent with each successive re-infection (Supplementary Figure S12D).

### Reduced therapeutic capacity of Symbioflor-2 is in part ascribed to control by innate effector cells

**Figure 7:**
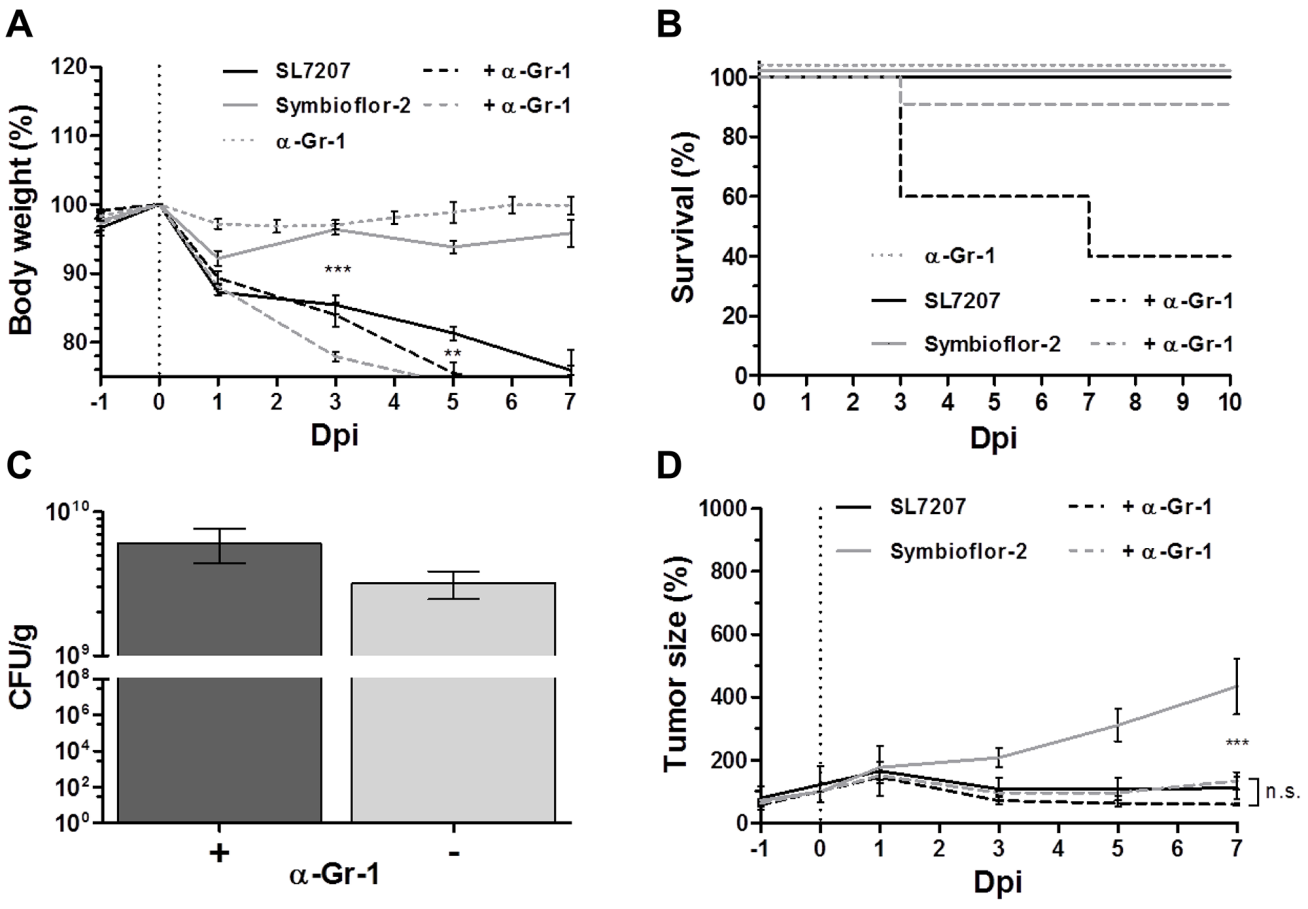
Gr-1 depletion improves tumor therapeutic effects with Symbioflor-2 to the extent of SL7207 RenCa tumor bearing mice received a daily injection of 25 μg anti-Gr-1 mAb (α-Gr-1) i.p. **A.** Progression in host body weight of Gr-1 depleted mice following infection. **B.** Mortality rate of Gr-1 depleted mice over the course of infection. **C.** RenCa tumor colonization 48 hpi with and without depletion of Gr-1 **D.** RenCa tumor development in Gr-1 depleted mice over the course of infection. N=5-8. Mean ± SEM.

One may speculate on the differential anti-tumor effects between *E. coli* probiotics and SL7207. Neutrophilic granulocytes have been described to be important for infection control [48, 72, 73]. In the tumor, they were observed to reside in close proximity to the probiotics. Thus, we depleted Gr-1 positive cells and evaluated the therapeutic outcome. Gr-1 depletion per se was well tolerated. However, in combination with infection, mice were seriously affected. Regardless infection with Symbioflor-2 or SL7207, the body weight loss of the wild-type host was equally progressive upon Gr-1 depletion (Figure 7A). Furthermore, infection under these conditions was accompanied by an increased mortality rate. Altogether a small proportion of mice succumbed to infection with Symbioflor-2 while only 40% survived the infection with SL7207 (Figure 7B). Although below significance, tumor colonization with Symbioflor-2 was slightly increased upon Gr-1 depletion, suggesting more widespread colonization, and supporting the concept of neutrophil control (Figure 7C).

We asked if these cells restrain the therapeutic effect of Symbioflor-2. RenCa tumor development remained similar for SL7207 infection regardless of Gr-1 depletion (Figure 7D). Surprisingly, the same level of tumor retardation was achieved by Symbioflor-2 infection when Gr-1^+^ cells were depleted (Figure 7D). Thus, the constraint of Symbioflor-2 by cells of the innate immune system limited the therapeutic efficacy of the *E. coli* probiotic.

**Figure 8:**
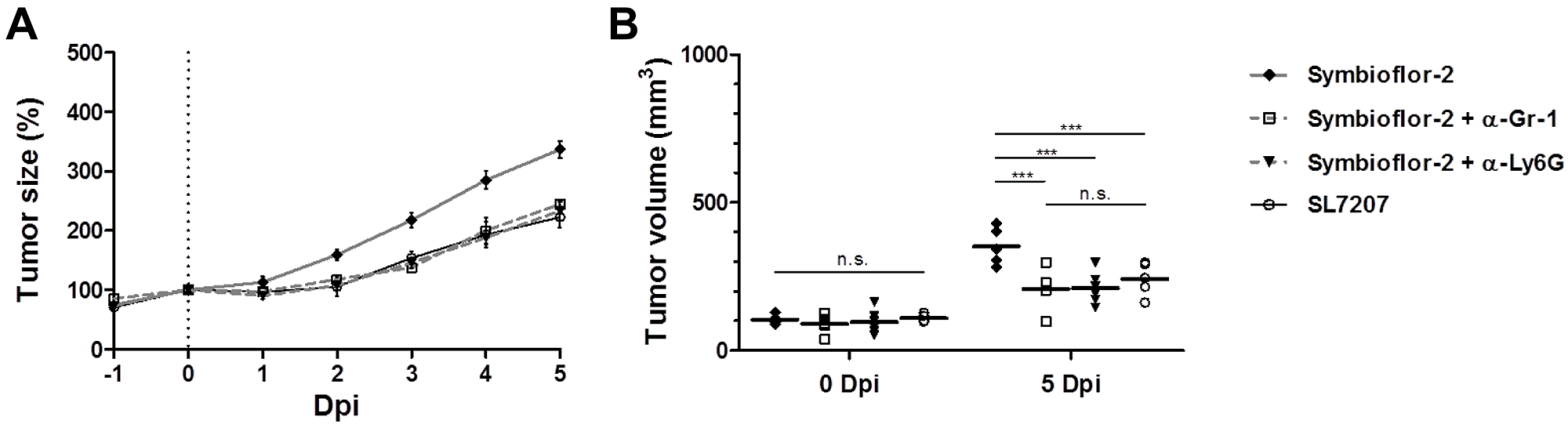
Specific depletion of Ly6G highlights neutrophils as a restraining factor for Symbioflor-2 mediated tumor therapy RenCa tumor bearing mice received a daily injection i.p. of 25 μg anti-Gr-1 mAb (RB6-8C5) or anti-Ly6G mAb (1A8). **A.** Tumor development in mice subjected to infection with SL7207 or Symbioflor-2 alone or in combination with anti-Gr-1 mAb (α-Gr-1) or anti-Ly6G mAb (α-Ly6G). **B.** Comparable endpoint tumor volume between SL7207 infection and combination treatments Symbioflor-2/ α-Ly6G and Symbioflor-2/ α-Gr-1. N=5. Mean ± SEM.

To validate our finding and substantiate evidence against neutrophils as a responsible candidate we analyzed the effect of Symbioflor-2 infection on RenCa tumor development in mice depleted of Ly6G, a more specific marker of neutrophils [74]. RenCa tumor growth was equally retarded in Symbioflor-2 infected mice upon treatments using Ly6G mAb and Gr-1 mAb (Figure 8A, 8B). This result highlights neutrophils as a restraining factor for the intrinsic therapeutic effects of Symbioflor-2.

## DISCUSSION

In the present work we validated the safety parameters of probiotic *E. coli* upon systemic infection as well as the intrinsic cancer therapeutic efficacy using the murine transplantable tumor models CT26 and RenCa. As standard, we used the *Salmonella* variant SL7207 *(ΔaroA*, *hisG^−^*) which is frequently used as vaccine carrier [75–78], and has been deployed in many of our previous studies [21, 47–49]. We found that *E. coli* probiotics generally display a greater specificity of tumor colonization and an improved safety profile. However, therapeutic efficacy was inferior compared to SL7207, which could be partially explained by control of the probiotics by Gr-1^+^ or Ly6G^+^ cells. Depletion of such innate immune cells strongly enhanced the intrinsic therapeutic efficacy of Symbioflor-2.

We based our study on the assumption that bacteria which had extensively proven their safety as oral probiotics would also sustain safety when applied systemically for tumor therapy. This turned out to be only partially correct. EcN together with all strains that comprise Symbioflor-2 displayed efficient tumor targeting as well as superior tumor specificity compared to SL7207. Contrary to SL7207, *E. coli* probiotics appear to be cleared very quickly from spleen and liver leaving only the tumor colonized as immune privileged site. The rapid clearance of probiotics from healthy organs accounts for the superior tumor specificity of probiotics and suggests an effector mechanism of the innate immune system although this was not clearly reflected in our *in vitro* assays on phagocytosis and complement sensitivity. However, these assays reflect *in vivo* conditions only to a very limited extent.

The fast clearance of the bacteria from liver and spleen most likely explains the excellent health status of mice infected by the probiotics. They tolerated even 100-fold increased doses of bacteria without apparent manifestation of pathology. Nevertheless, we encountered pathogenic potential. Probiotic Symbioflor-2 bacteria were invasive for gnotobiotic mice upon oral application (data not shown). Similar results had been obtained for EcN earlier [79]. However, toxicity of EcN in the immune compromised Rag1^−/−^ mice proved a revelation, given that Symbioflor-2 bacteria did not cause a similar aberrant clinical phenotype in these mice. The reason for the discrepancy between these strains remains unclear. The phenotype is unlikely ascribed to the genotoxin colibactin in EcN as the mutant strain *Δclb*, in which this toxin had been deleted, was as pathogenic as the parental bacteria. Genomic comparison revealed differences between EcN and Symbioflor-2 but also among individual strains of Symbioflor-2 (Supplementary Table S1). Such differences may in part explain for an increase of virulence of EcN under immunocompromised conditions. However, this would remain purely speculative at this point. Overall, our premises for the high safety standard for probiotics are only valid for Symbioflor-2 strains and not for EcN as long as its pathogenicity is not clarified. Therefore, our focus remained on Symbioflor-2.

The exquisite safety features were not accompanied by an equivalent therapeutic potency. Although both probiotics were able to clear most CT26 tumors similar to SL7207, Symbioflor-2 and EcN hardly had an effect on the growth rate of RenCa tumors. In contrast, SL7207 severely retarded the growth of this tumor. Even altering the infection parameters, such as increasing the inoculum of Symbioflor-2 by 100-fold did not install a missing therapeutic effect.

Interestingly, we found that the lower therapeutic capacity of these bacteria was ascribed to their sensitivity to Gr-1^+^ cells of the innate immune system. We have previously observed that the depletion of such cells, which mainly consist of neutrophilic granulocytes, restrains the efficacy of BMTT [48]. Recent reports describe a diversity of Gr-1^+^ cells that are involved in infection control (e.g. inflammatory monocytes, subpopulations of CD8^+^ T cells as well as neutrophils) [74, 80, 81]. Therefore, ascribing the therapeutic restraint to neutrophils required confirmation. Similar results were obtained using the more specific depleting mAb anti-Ly6G (1A8). This provides evidence that therapeutic efficacy of Symbioflor-2 indeed can be enhanced through concurrent depletion of neutrophils alone.

One explanation for the enhanced therapeutic capacity in absence of these protective cells might be un-prohibited access to viable areas of the tumor where they would facilitate a more extensive therapeutic effect [82, 83]. Alternatively, it might be caused by increased numbers of bacteria colonizing the tumor as result of more widespread dissemination. However, no significant increase in tumor CFU counts was obtained with Symbioflor-2. Importantly, under such conditions, Symbioflor-2 exhibited the same therapeutic potency towards RenCa as seen with SL7207. However, it also increased the pathogenic burden imposed on the mice. Again, this supports an unfavorable dependence between safety and therapeutic benefit. Some mice even succumbed under these infection conditions.

Our experiments also confirmed the two-phase nature of the therapeutic mechanism. The first consists of effector mechanisms of the innate immune system which are also observable in Rag1^−/−^ mice deprived of adaptive immune components. Most likely this first effector phase includes the secretion of TNFα which facilitates hemorrhage in the tumor, and the formation of large necrotic regions providing ideal conditions for the influx and proliferation of bacteria in the same phase [46]. Hemorrhage and necrosis formation may per se be responsible for initial growth retardation. The lower level of induction by Symbioflor-2 might in part explain the contrasting effects between SL7207 and the probiotics observed on RenCa tumors.

The second phase is dominated by tumor-specific T cells, - mainly CD8 positive cytotoxic T cells [50, 84–86]. Interestingly, such cells are already present in the tumor-bearing mice before BMTT. Even so, tumors are able to grow despite their presence. Only upon inoculation with bacteria, such cells become sufficiently activated and are able to counteract tumor growth, causing growth retardation and, in some cases, even complete rejection. Thus, the bacteria act as a strong adjuvant. It remains unclear why exactly the activation by SL7207 and its therapeutic efficiency is sustained, whereas application of probiotics only results in transient reduction in some of the CT26 tumors.

Symbioflor-2 strains display the ability to colonize distinct solid tumors with high specificity and low side effects upon systemic inoculation. However, the excellent safety profile is contrasted by an inferior therapeutic efficacy. Nevertheless, basing a bacterial therapy along an adjuvant effect alone might prove unfeasible [87–90]. Symbioflor-2 needs to undergo recombinant strengthening to gain efficiency, either by supplementing the bacteria with additional virulence factors and/ or equipping them with expression and secretion systems that ensure delivery of therapeutic molecules to the target tumor. Thus, feasible exploitation remains as a specific bio-vehicle in context of systemic BMTT.

## EXPERIMENTAL PROCEDURES

### Ethics statement

All animal experiments were performed according to the guidelines of the German Law for Animal Protection and with permission by the local ethics committee and authority LAVES (Niedersächsisches Landesamt für Verbraucherschutz und Lebensmittelsicherheit). The animal permission was issued under number: 33.9-42502-04-12/0713.

### Animal models and tumor development

All mice were kept in IVC cages under SPF conditions. Six to eight weeks old female BALB/c mice (Janvier) were inoculated subcutaneously with 100 μl 5×10^5^ CT26 cells (ATCC CRL-2638) or 1.5×10^6^ RenCa cells (ATCC CRL-2947) in PBS into the flank and allowed to establish over 8-10 days. When reaching a size of 100-150 mm^3^ the mice were regrouped according to mean tumor volume, and subjected to infection. Regular caliper measurements in two dimensions allowed for volume calculations using the formula V = π/6 x (h x w^2^), wherein “h” = height and “w” = width. “Tumor size” denotes a measure of tumor development standardized to tumor volume at 0 dpi. Rag1^−/−^ mice were bred in house and females at six to ten weeks of age were deployed. To achieve neutropenic conditions, we adhered to the protocol of Westphal et al. [48] In brief: mice received repeated doses of 25 μg monoclonal rat anti-Gr-1 (RB6-8C5) or 25 μg monoclonal rat anti-Ly6G (1A8) i.p. Depletion was confirmed by flowcytometry.

### Infection procedures and bacterial recovery from tissues

Symbioflor-2 strains G1/2, G3/10, G4/9, G5, G6/7, and G8 (DSM16441, DSM16443, DSM16444, DSM16445, DSM16446, and DSM16448, respectively) [45], and *E. coli* Nissle 1917 (EcN, DSM6601) otherwise commercialized as Mutaflor were kindly provided by Prof. Florian Gunzer (TUD, Dresden, Germany) and Dr. Kurt Zimmermann (SymbioPharm, Herborn, Germany). EcN *Δclb* was kindly provided by Prof. Rolf Müller at HIPS, Saarbrücken, Germany. *Salmonella* Typhimurium SL7207 (*hisG*^−^, *ΔaroA*) [91], ATCC14028 (denoted “S.T. WT”), and *Pseudomonas aeruginosa* PA14 were deployed from our stock collection. Strains were all grown in LB medium overnight (ON) at 37°C, subcultured 1:100 and finally harvested at midlog phase, washed and adjusted to appropriate inoculum in pyrogen-free PBS. The Symbioflor-2 cocktail represents a pool of six individual strains combined at an equal ratio. Plating controls were performed to verify the inocula. Bacterial suspensions of 100 μl per mouse at a standard dose of 5×10^6^ were delivered intravenously via the lateral tail vein. At time points of interest, mice were sacrificed using CO_2_. Final blood collection for isolation of serum was performed via heart puncture, and for direct plating instantly mixed 1:1 with 0.1% Heparin (in PBS). Tumors, livers and spleens, were aseptically isolated and transferred to 1 ml sterile PBS containing 0.1% (v/v) Triton X-100. For determination of CFU, tissues were homogenized using a gentleMAX dissociator (Miltenyi Biotec), plated in serial dilutions, and incubated ON at 37°C.

### Non-invasive *in vivo* imaging

To visualize the live distribution of bacteria over a certain time frame in the same mouse, the plasmid pHL304 encoding the *luxCDABE operon* from *Photorhabdus luminescens* facilitating constitutive expression of bioluminescent Lux was transformed with *E. coli* G1/2 via electroporation [33]. Prior to image acquisition via the IVIS200 System (Calipers), mice were anaesthetized with 2% Isoflurane using the XGI-8 gas system (Calipers). Image analysis was performed using the Living image software (Xenogen).

### Adoptive transfer experiments

Spleenocytes isolated from infected or naive BALB/c mice were suspended in IMDM medium and erythrocytes lysed in ACK buffer. CD4^+^ and CD8^+^ T cell populations were purified using negative isolation kits (Invitrogen), and verified using flow cytometry by staining for CD3, CD4, CD8, and CD19. The isolated T cell populations were free of B cells and contrasting T cell population. For reconstitution experiments, 3×10^6^ T cells were adoptively transferred via i.v. injection into Rag1^−/−^ mice.

### Histology

Upon isolation, specimens were fixed with 4% (v/v) formalin for 24 – 48 h, embedded in paraffin. Approximately 3 μm thick sections were stained with hematoxylin/ eosin according to standard laboratory procedures. Immuno-histo-chemical staining was performed using the following antibodies: goat-anti-*E. coli* (USBiological, E3500-06J), rabbit-anti-Ki-67 (Neo Makers, RM-9106-S), and rabbit-anti-pimonidazole (HP3-100 kit, Hydroxyprobe inc.) and DAB (3,3-Diaminobenzidine Zytomed Systems DAB530) as chromogen. Hematoxylin was used for counterstaining. Sections were analyzed by light microscopy blinded to the experimental groups.

### Biofilm analyses

The capacity to form biofilm *in vitro* was determined by spiking biomass onto biofilm plates containing LB medium, low salt, Congo red, and coomassie brilliant blue. Plates were grown at 30°C for 20 days (modified from Crull et al.) [51]. To quantify static biofilm on a plate, cultures were allowed to grow for 48 hpi at 37°C, before staining with 0.1% Crystal violet. Following several washing steps to removed planktonic bacteria, de-staining with 95% ethanol ensured a suspension ready for absorbance measurement at OD_600_.

### Electron microscopy

To evaluate biofilm formation *in vivo*, tumors were isolated 48 hpi and fixed in 5% formaldehyde and 2% glutaraldehyde. Upon fixation the tumor was cut into 5 mm cubes, contrasted in aqueous osmium tetroxide, dehydrated with a graded series of acetone, and infiltrated with an epoxy resin (Spurr 1969) [51, 92]. Ultrathin sections were cut using a diamond knife, picked up with formvar coated grids and counterstained with uranyl acetate and lead citrate. Samples were analyzed with a TEM910 transmission electron microscope (Carl Zeiss, Oberkochen) at an acceleration voltage of 80 kV at calibrated magnifications. Images were recorded digitally with a Slow-Scan CCD-Camera (ProScan, 1024 × 1024, Scheuring, Germany) with ITEM-Software (Olympus Soft Imaging Solutions, Münster, Germany).

### Phagocytosis and intracellular survival

An *in vitro/ ex vivo* gentamicin survival assay was used to determine the relative phagocytic uptake and intracellular survival of bacteria. In a first instance, the macrophage cell line J774 was used while later murine bone-marrow derived macrophages (BMDMs) were subject to our assays. BMDMs we acquired by differentiation of isolated precursors matured in presence of M-CSF over 10 days, before verified in FACS and used directly for the gentamycin assay. Bacterial suspensions were added at an MOI of 15 (5×10^6^ CFU/ 1×10^5^ macrophages) and incubated for 1 h at 37°C. Upon washing, treatment with 100 μg/ml gentamicin followed to eradicate extracellular bacteria. Mechanical detachment was followed by plating in serial dilutions to determine the CFU count of intracellular shielded bacteria. Incubation until 17 h post gentamicin treatment and plating served to determine the intracellular survival.

### Complement sensitivity

Peripheral blood samples were drawn from healthy human volunteers, or dogs. Serum was isolated using Microvette serum tubes (Sarstedt), a proportion of the serum was heat inactivated at 56°C for 2 h. Bacterial suspensions of 1×10^7^ in 100 μl were challenged 1:1 with untreated or heat inactivated serum for 30 min incubation at 37°C, upon which the bacteria were plated in serial dilutions for CFU counting to determine susceptibility to complement lysis.

### Statistical analyses

Significance between two groups was determined using the nonparametric Mann-Whitney test, while one-way analysis of variance (ANOVA) with Bonferroni posttest was used to compare two or more groups. Significance levels of p<0.05, p<0.01, or p<0.001 were denoted with asterisks: *, **, and ***, respectively.

## SUPPLEMENTARY DATA FIGURES AND TABLE


