# Supplementary material for: Therapy of solid tumors using probiotic Symbioflor-2 – restraints and potential
Source: Oncotarget. 2016 Mar 10;7(16):22605–22. doi: 10.18632/oncotarget.8027 (PMC5008385; doi:10.18632/oncotarget.8027)
Supplement: Supplementary file 1 [file oncotarget-07-22605-s001.pdf]

SUPPLEMENTARY DATA

METHODS

All genes and gene loci presented in Table S1 were verified either by browsing the annotated genomes in GenDB 2.4 (www.gendb.cebitec.uni-bielefeld.de/cgi-bin/login.cgi) or by performing homology searches using the BLASTN algorithm [10] in GenDB 2.4 and/ or at the National Center for Biotechnology Information (http://blast.ncbi.nlm.nih.gov/Blast.cgi).

RESULTS

Genomic comparison between Symbioflor-2 and EcN

The genomes of all six Symbioflor-2 *E. coli* strains are slightly smaller (4.51 Mb - 4.98 Mb) compared to the EcN genome (5.05 Mb). In addition these probiotics exhibit genetic differences. Review **Supplementary Table S1** for selected examples. In brief: EcN strongly produces curli fimbriae [1, 2] while of the Symbioflor-2 strains only *E. coli* G3/10 bares a complete *csg* locus. EcN also contains the type 1 fimbriae operon and the *iha* encoded bifunctional enterobactin receptor/ adhesin. These features are not found in all Symbioflor-2 *E. coli* [3]. On the other hand, *E. coli* G3/10 is the only Symbioflor-2 *E. coli* to harbor the *mrk* locus [3], encoding type 3 fimbriae, a feature which EcN does not have. The large gene that encodes UpaG, a member of the trimeric autotransporter family of adhesins [4], is only present in the EcN genome. EcN also carries genetic information for all six iron uptake systems aerobactin, *E. coli* hemin uptake system, enterobactin, ferric dicitrate transport system, salmochelin and yersiniabactin [2]. Heterogeneity amongst the probiotics is found regarding toxins colicin-Ib, colicin S4, microcin H47, microcin M and microcin S [5, 6]. In the three Symbioflor-2 *E. coli* strains G1/2, G6/7 and G8 a complete *hly* locus encoding *E. coli* α-hemolysin is present [3], resulting in a weakly hemolytic phenotype [8]. In a recent volunteer study, exactly these strains colonized the human gut persistently [7]. EcN is negative for *E. coli* α-hemolysin. The weakly hemolytic Symbioflor-2 *E. coli* also possess a truncated *eib* locus, encoding genes responsible for nonimmune immunoglobulin-binding activities of *E. coli,* also conferring serum resistance [9]. EcN lacks the *eib* operon. All Symbioflor-2 *E. coli* and EcN have the genes necessary to synthesize glutamate decarboxylase which is important for unscathed transit through the stomach and successful colonization of the host by enteric bacteria. EcN has the serotype O6:K5:H1 [11] and displays a semi rough LPS phenotype due to a nonfunctional *wzy* gene, that also impacts the strain's resistance to serum [12]. In contrast, with the exception of *E. coli* G3/10 (LPS serotype O:35,129) and G8 (LPS serotype O:169), the Symbioflor-2 *E. coli* are rough and nonmotile [3].

REFERENCES

1. Monteiro C, Saxena I, Wang X, Kader A, Bokranz W, Simm R, Nobles D, Chromek M, Brauner A, Brown RM, Römling U. Characterization of cellulose production in *Escherichia coli* Nissle 1917 and its biological consequences. Environ Microbiol. 2009; 11:1105–16.
2. Grozdanov L, Raasch C, Schulze J, Sonnenborn U, Gottschalk G, Hacker J, Dobrindt U. Analysis of the genome structure of the nonpathogenic probiotic *Escherichia coli* strain Nissle 1917. J Bacteriol. 2004; 186:5432–41.
3. Wassenaar TM, Zschüttig A, Beimfohr C, Geske T, Auerbach C, Cook H, Zimmermann K, Gunzer F. Virulence genes in a probiotic *E. coli* product with a recorded long history of safe use. Eur J Microbiol Immunol. 2015; 5:81–93.
4. Valle J, Mabbett AN, Ulett GC, Toledo-Arana A, Wecker K, Totsika M, SChembri MA, Ghigo JM, Beloin C. UpaG, a new member of the trimeric autotransporter family of adhesins in uropathogenic *Escherichia coli*. J Bacteriol. 2008; 190:4147–61.
5. Patzer SI, Baquero MR, Bravo D, Moreno F, Hantke K. The colicin G, H and X determinants encode microcins M and H47, which might utilize the catecholate siderophore receptors FepA, Cir, Fiu and IroN. Microbiology. 2003; 149:2557–70.
6. Zschüttig A, Zimmermann K, Blom J, Goesmann A, Pöhlmann C, Gunzer F. Identification and characterization of microcin S, a new antibacterial peptide produced by probiotic *Escherichia coli* G3/10. PLoS One. 2012; 7:e33351.
7. Wassenaar TM, Beimfohr C, Geske T, Zimmermann K. Voluntarily exposure to a single, high dose of probiotic *Escherichia coli* results in prolonged colonisation. Benef Microbes. 2014; 5:367–75.
8. Willenbrock H, Hallin P, Wassenaar T, Ussery D. Characterization of probiotic *Escherichia coli* isolates with a novel pan-genome microarray. Genome Biol. 2007; 8:R267.
9. Sandt C, Hill C. Four Different Genes Responsible for Nonimmune Immunoglobulin-Binding Activities within a Single Strain of *Escherichia coli*. Infect Immun. 2000; 68:2205–14.
10. Altschul S, Madden T, Schäffer A, Zhang J, Zhang Z, Miller W, Lipman D. Gapped BLAST and PSI-BLAST:

a new generation of protein database search programs. Nucleic Acids Res. 1997; 25:3389–402.

11. Blum G, Hacker J, Marre R. Properties of *Escherichia coli* strains of serotype O6. Infection. 1995; 23:234–6.

12. Grozdanov L, Zahringer U, Blum-Oehler G, Brade L, Henne A, Knirel YA, Schombel U, Schulze J, Sonnenborn U, Gottschalk G, Hacker J, Rietschel ET, Dobrindt U. A Single Nucleotide Exchange in the wzy Gene Is Responsible for the Semirough O6 Lipopolysaccharide Phenotype and Serum Sensitivity of *Escherichia coli* Strain Nissle 1917. J Bacteriol. 2002; 184:5912–25.

SUPPLEMENTARY FIGURES AND TABLE

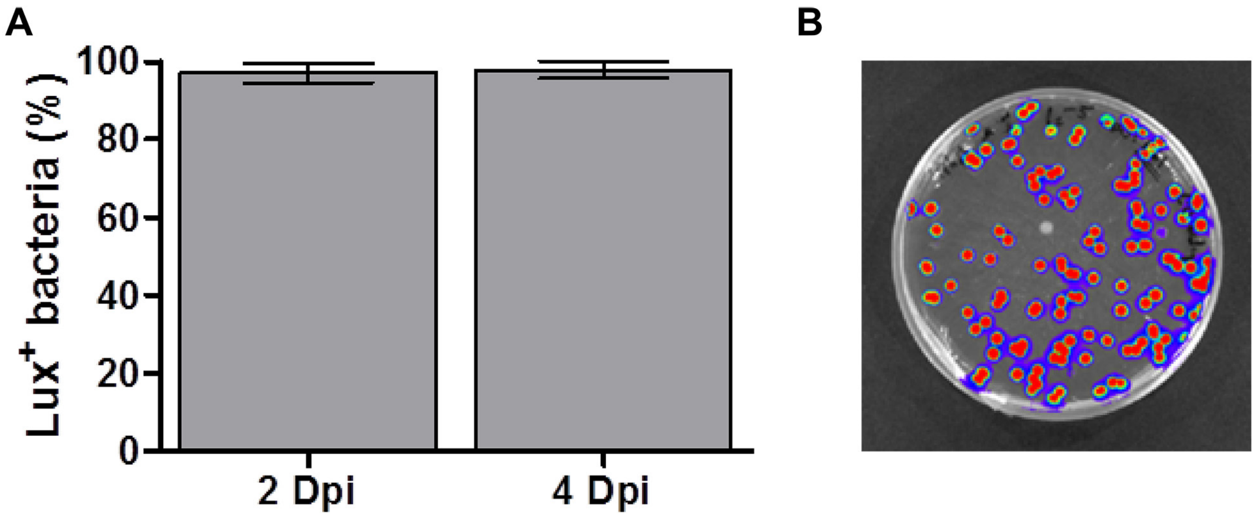

Supplementary Figure S1: *In vivo* stability of G1/2 transformants. Plasmid pHL304 was transformed into strain G1/2 to confer constitutive Lux expression for IVIS analysis. Stability of the G1/2 transformants was determined via plating of CT26 tumors on selective agar on day 2 and 4 p.i. **A.** The difference in yield between Amp+ transformants and unspecific LB agar was used to calculate % Lux positive G1/2 bacteria. **B.** Qualitative example of an IVIS-analyzed unspecific plating of a colonized tumor harvested day 4 p.i. N=3. Mean ± SD.

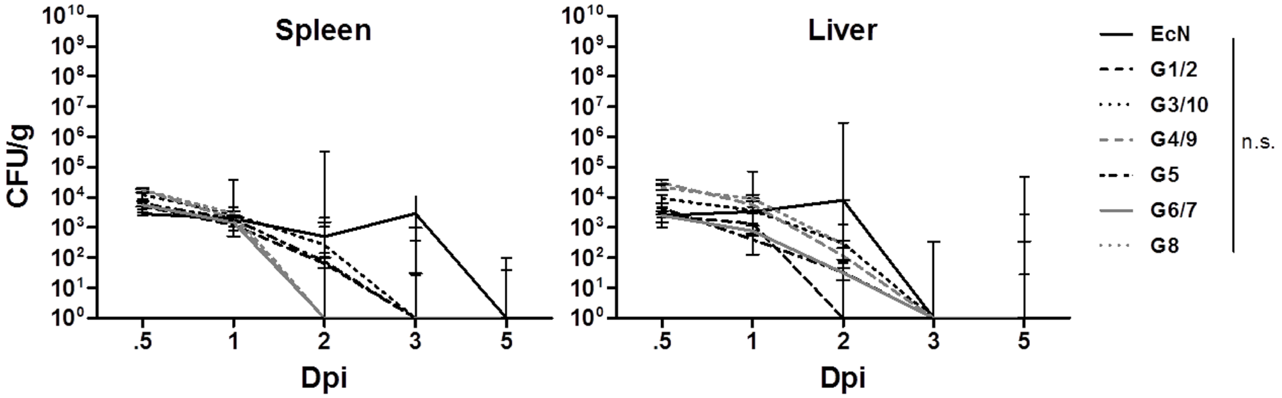

Supplementary Figure S2: Similar kinetic of colonization in liver and spleen among *E. coli* probiotics**.** Spleen and liver was harvested from infected CT26 tumor bearing mice before homogenized and plated in serial dilutions to determine CFU counts. All *E. coli* strains tested are gradually cleared from these compartments. N=5. Median ± range.

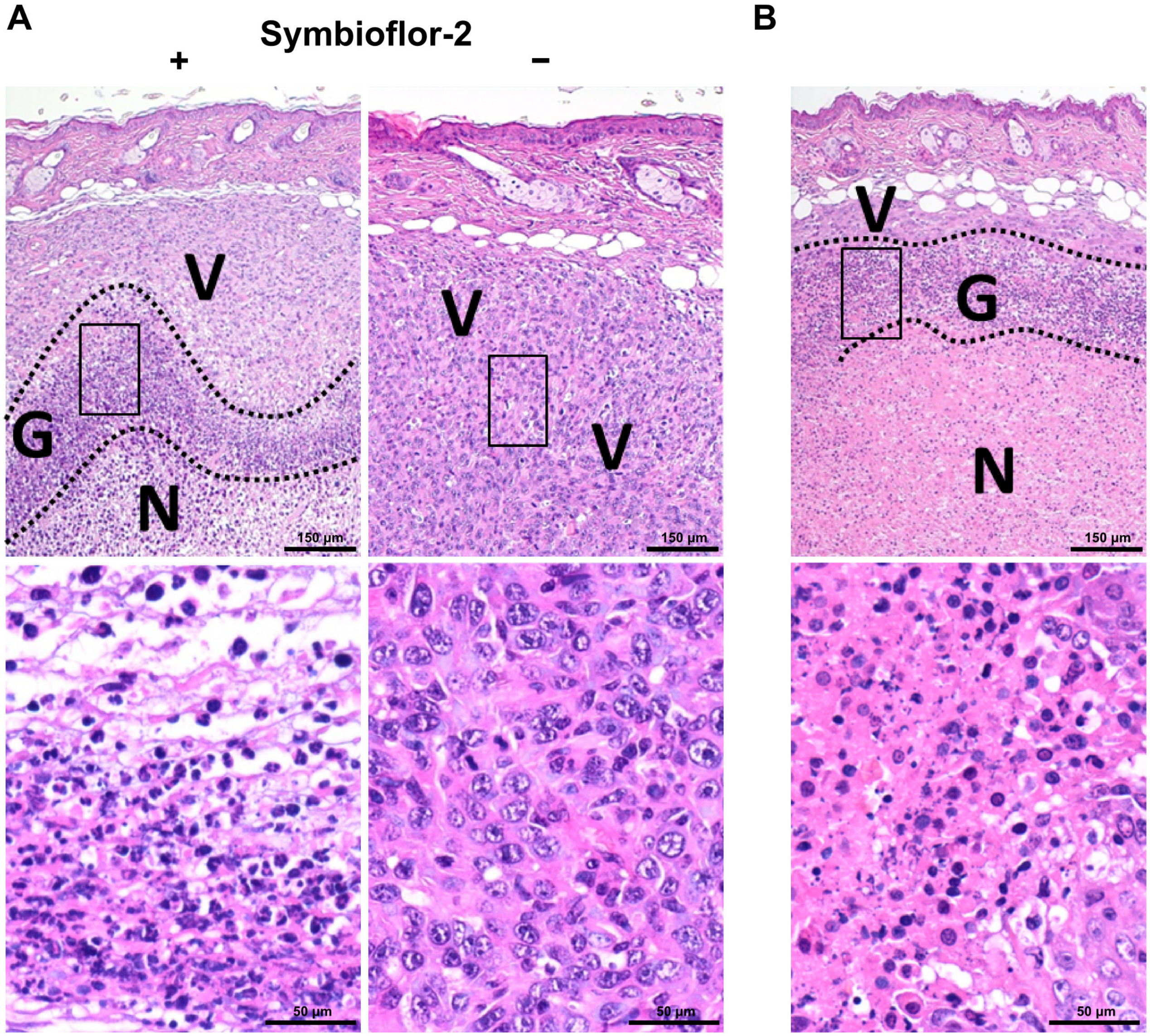

Supplementary Figure S3: Infection supports necrosis formation, and granulocytic accumulations in the interphase towards viable tissue. Tumor cross sections were subjected to classical histological analysis 48 hpi with Symbioflor-2. **A.** HE staining of infected and uninfected (CTRL) CT26 tumor cross sections at 10x magnification (top row) and 40x magnification of highlighted regions (bottom row). High prevalence of cells with granulocytic morphology (G) in the interface between necrotic (N) and viable (V) tumor tissue. **B.** HE staining of infected RenCa tumor cross sections reveal a similar histological profile to CT26. Representative images are shown. N=3.

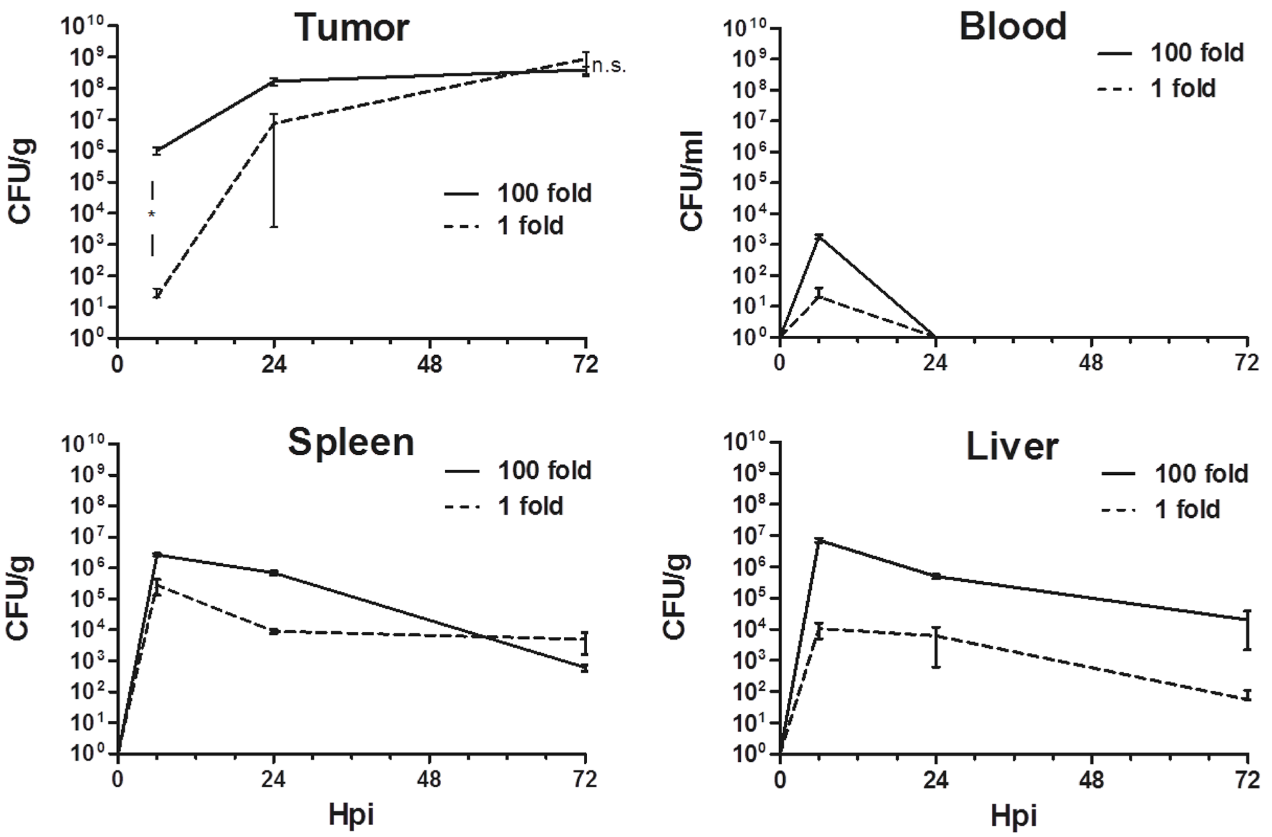

Supplementary Figure S4: Colonization profile upon high dose Symbioflor-2 infection. CT26 tumor bearing mice were inoculated i.v. with Symbioflor-2 at a standard dose of $5x10^6$ Symbioflor-2 or 100-fold the dose ($5x10^8$). Kinetic profiles of colonization over 72 hpi were compared for the CT26 tumor, blood, spleen, and liver. N=4. Median ± range.

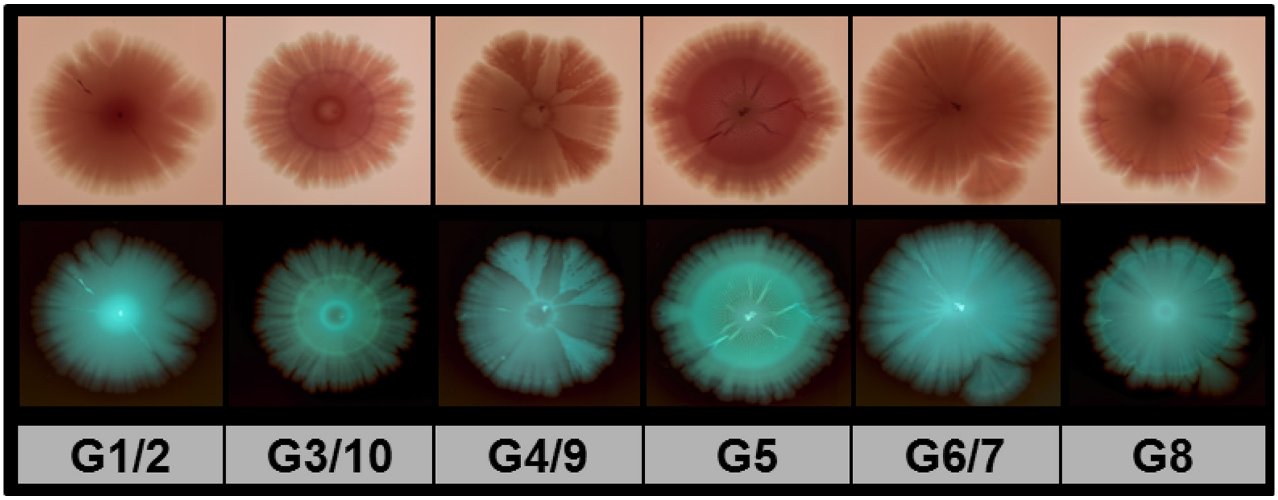

Supplementary Figure S5: Intrinsic capability of Symbioflor-2 strains to form biofilm under favorable conditions. *In vitro* biofilm forming capacity of individual Symbioflor-2 strains as determined by incubation of spiked agar plates at 30°C over 20 days. Images reveal an organized intricate network (biofilm). Bottom and top rows represent positively and negatively scanned biofilms, respectively.

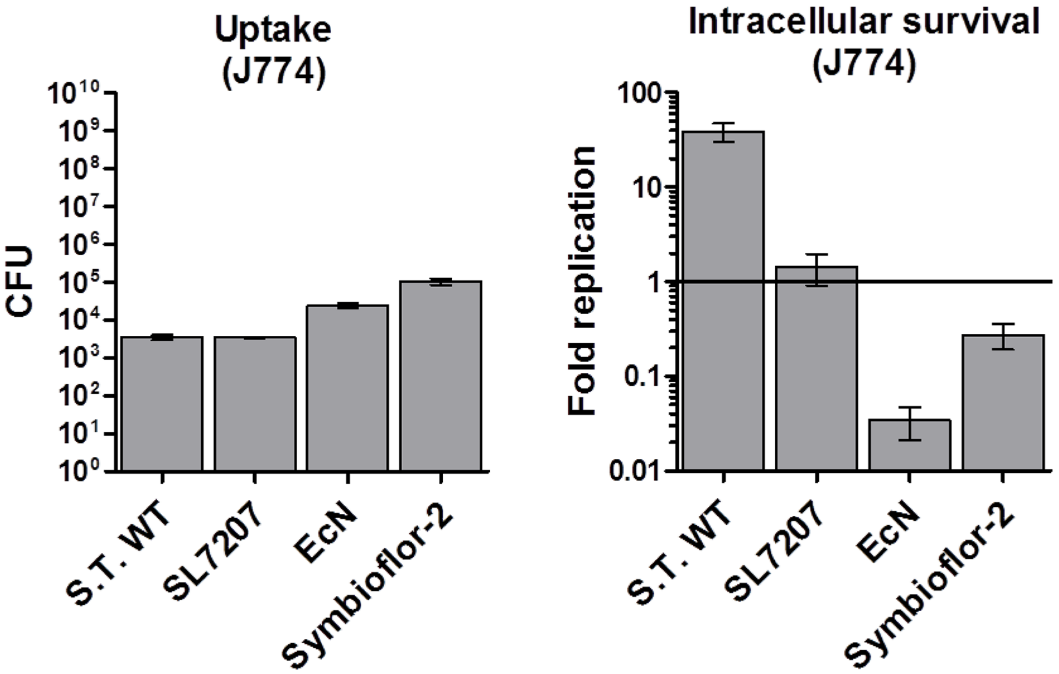

Supplementary Figure S6: Phagocytic uptake and intracellular survival in J774 macrophages. Bacterial uptake by ex vivo matured BMDMs, as determined through relative comparison of intracellular bacteria 1 hpi in a co-culture gentamicin survival assay (left panel). Intracellular survival/ replication of the bacteria in J774 macrophages as determined through intracellular CFU counts 18 hpi expressed relative to levels of uptake 1 hpi. N=5. Median ± range.

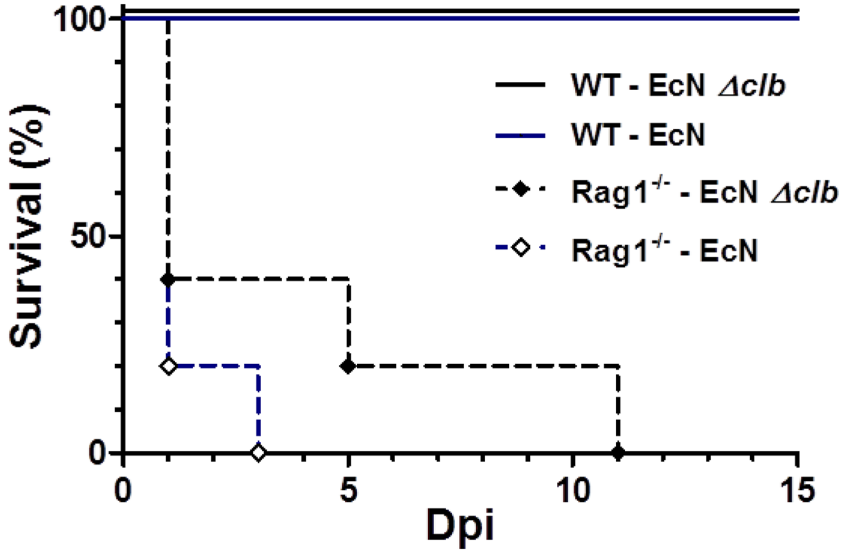

Supplementary Figure S7: EcN *Δclb* does not recover a detrimental infectious phenotype in Rag1$^{-/-}$ mice. Survival rate of WT and Rag1$^{-/-}$ mice infected with $5x10^6$ EcN or a colibactin negative mutant EcN *Δclb*. Absence of genotoxin colibactin does not recover a safe phenotype. N=5-8.

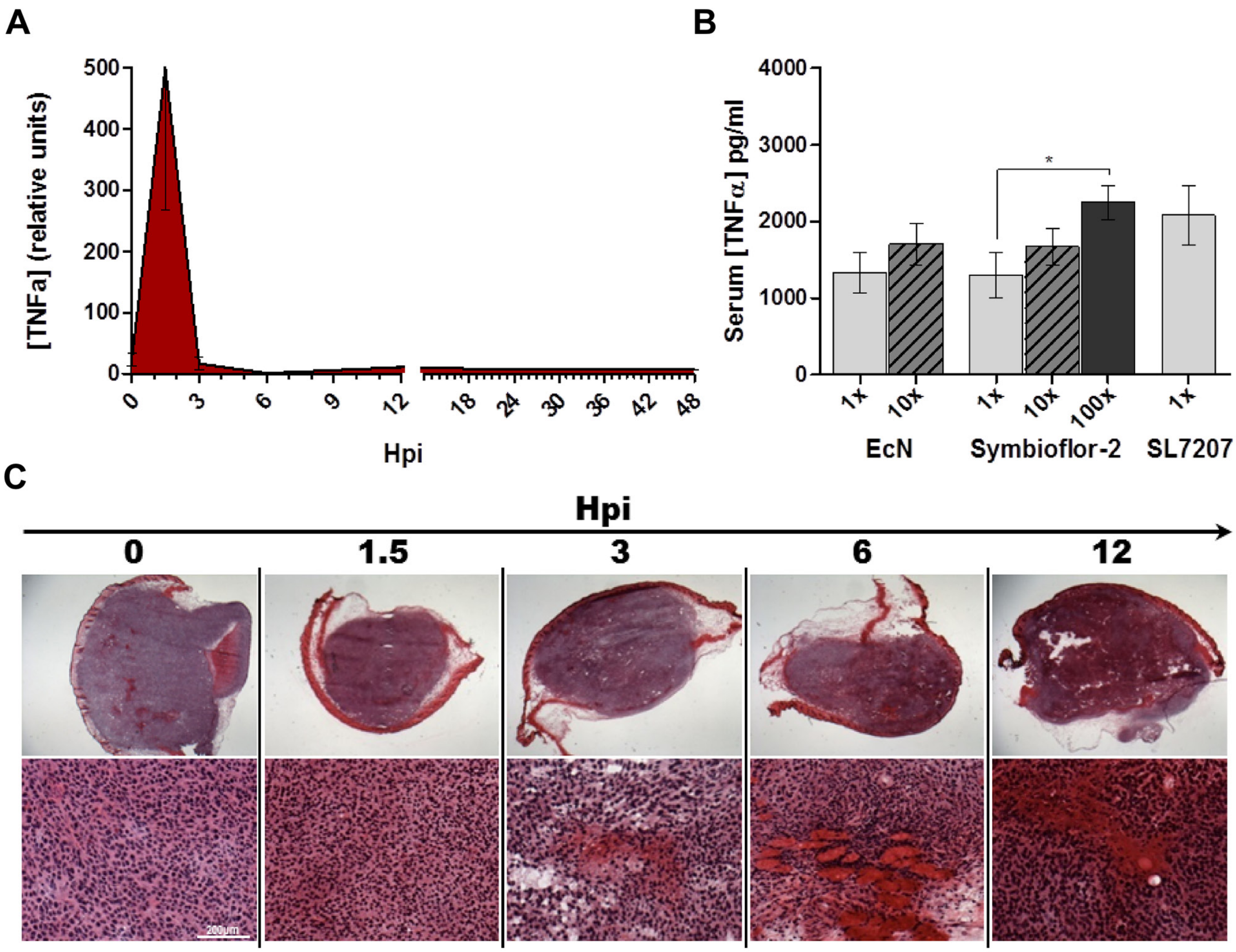

Supplementary Figure S8: Probiotic infection is accompanied by an early TNFα response and tumor hemorrhage. CT26 tumor bearing WT mice received a probiotic infection with *E. coli*, upon which serum and tumors were isolated at different time points. **A.** Kinetic of active serum TNFα as induced upon Symbioflor-2 infection and determined through a semi-quantitative bioassay using TNFα-sensitive C5F6 cells. **B.** Relative induction of systemic TNFα 1.5 hpi as determined using ELISA. **C.** HE staining of CT26 tumor cryo sections following probiotic infection show tumor hemorrhage and tissue deterioration within 12 hpi. 2.5x and 20x magnification in top and bottom rows, resp. N=3-5. Mean ± SEM.

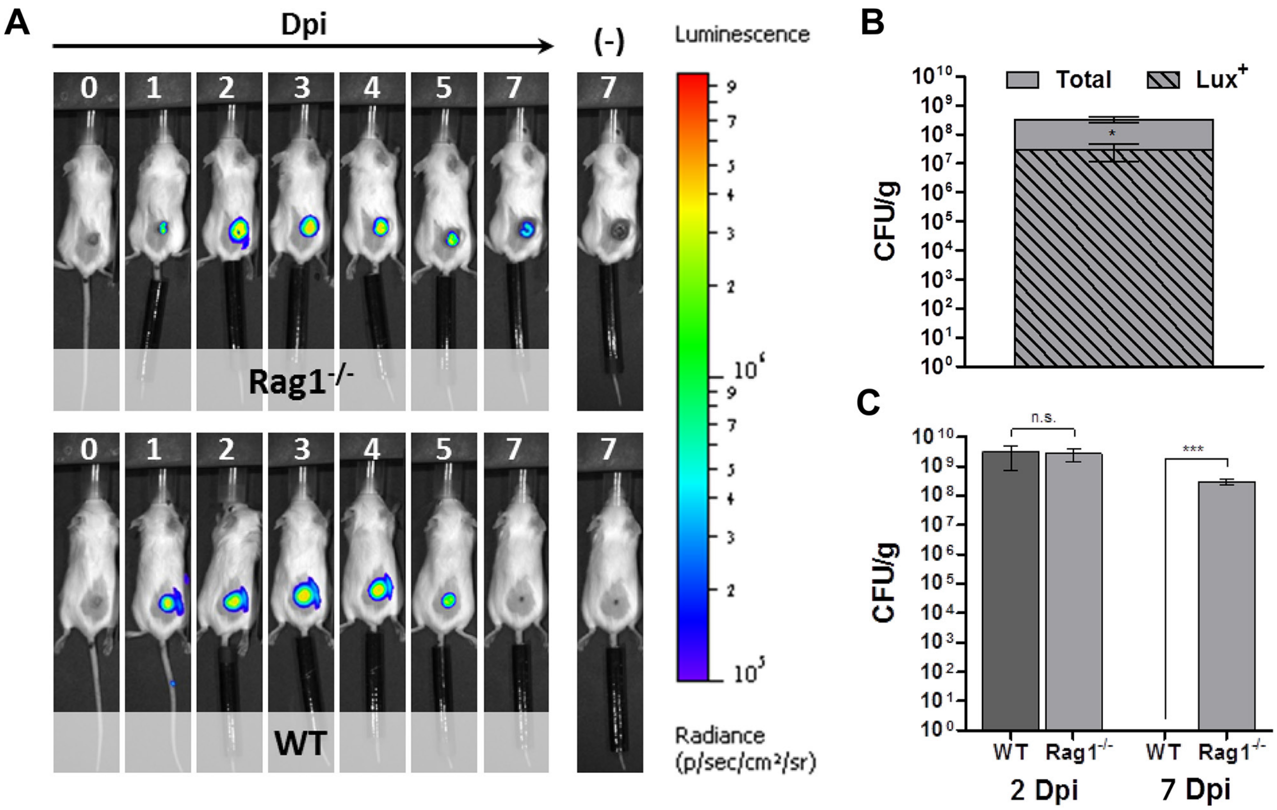

Supplementary Figure S9: Sustained tumor colonization in Rag1$^{-/-}$ mice. CT26 tumor bearing mice received an inoculum of 5x10^{6} probiotic *E. coli* transformed with a plasmid pHL304 encoding the *luxCDABE* operon. **A)** *In vivo* distribution kinetic in WT and Rag1$^{-/-}$ mice as analyzed using IVIS. **B)** IVIS control; Lux^{+} bacteria in the tumor at 7 dpi, as determined via specific and unspecific plating. **C)** Relative CFU in WT and Rag1$^{-/-}$ mice at 2 and 7 dpi showing persistent colonization in Rag1$^{-/-}$ mice. N=3-5. Mean ± SEM.

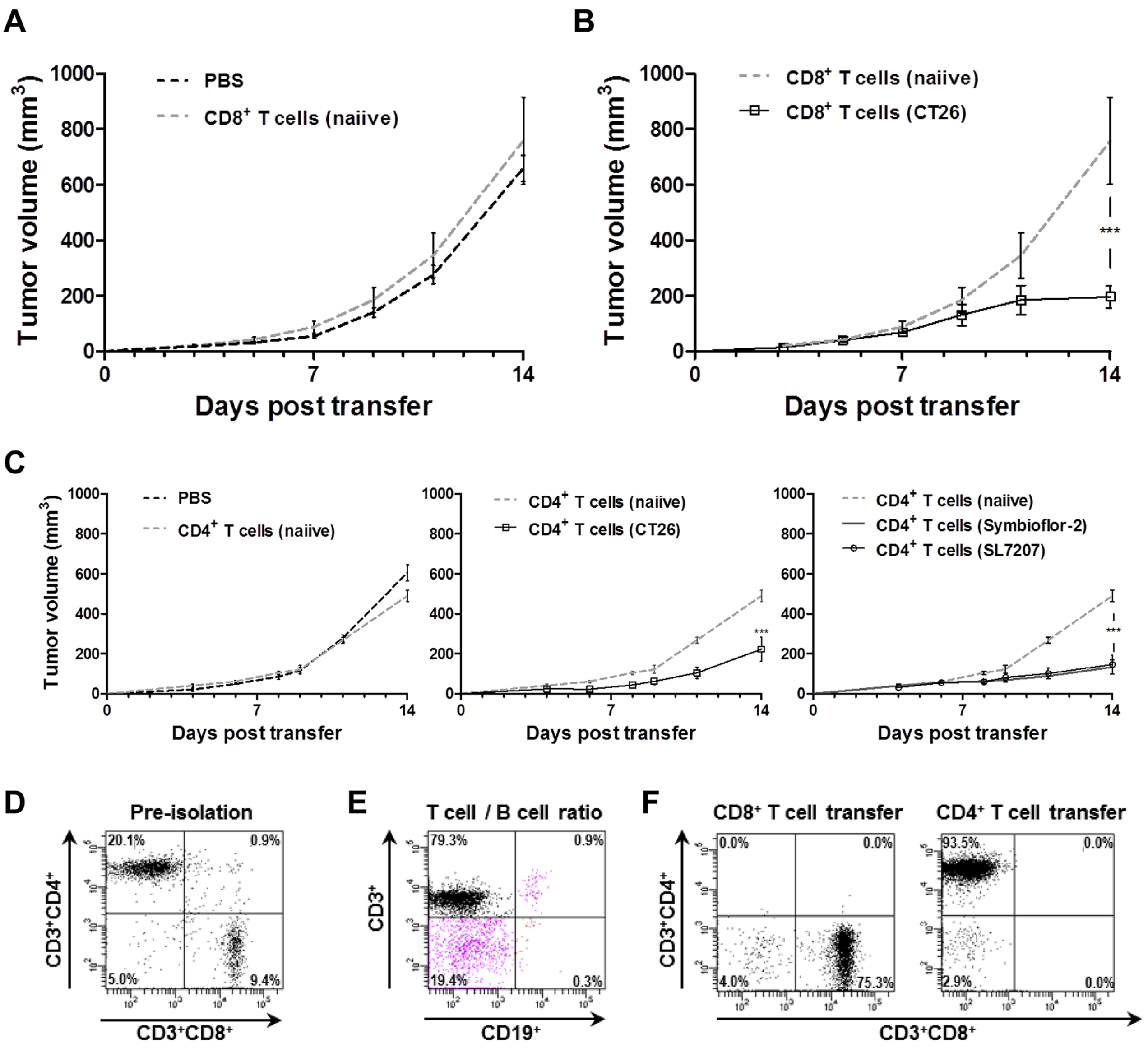

Supplementary Figure S10: Reconstitution of Rag1$^{-/-}$ mice with primed CD4^{+} and CD8^{+} T cells yields distinct tumor development profiles. WT mice having cleared CT26 tumors through infection were used as T cell donors for CT26 inoculated Rag1$^{-/-}$ recipients. CD4^{+} and CD8^{+} T cell populations were isolated from spleenocytes using standard isolation procedures and negative isolation kits. 3×10^{6} T cells were supplied i.v. per recipient **A.** Similar tumor development in Rag1$^{-/-}$ upon treatment with PBS or unstimulated CD8^{+} T cells. **B.** CT26 prestimulated CD8^{+} T cells per se retard tumor growth in Rag1$^{-/-}$ hosts. Preisolation conditions as indicated in the brackets. **C.** Reconstitution with CD4^{+} T cells does not yield significant tumor growth retardation. **D.** Fraction of CD4^{+} and CD8^{+} T cells in a sample pre negative isolation. **E.** Negligible proportion of B cells in the post isolation sample. **F.** Purity of CD4^{+}/ CD8^{+} T cells administered i.v.. N= 5-8. Mean ± SEM.

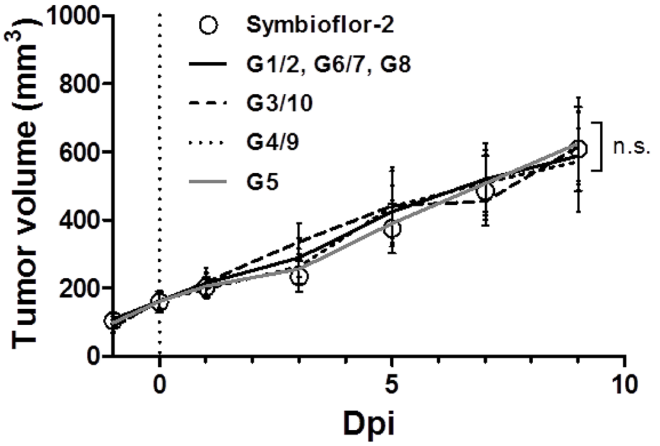

Supplementary Figure S11: Consistent RenCa tumor development profile among Symbioflor-2 strains. RenCa tumor growth was evaluated upon systemic infection with pooled Symbioflor-2 or individual strains. Due to close homology, also G1/2, G6/7, and G8, were equally pooled, administered and evaluated as one group. Total infection was kept consistent at $5x10^6$. Regardless the composition, all Symbioflor-2 infections yield consistent tumor development profiles. N= 5-8. Mean ± SEM.

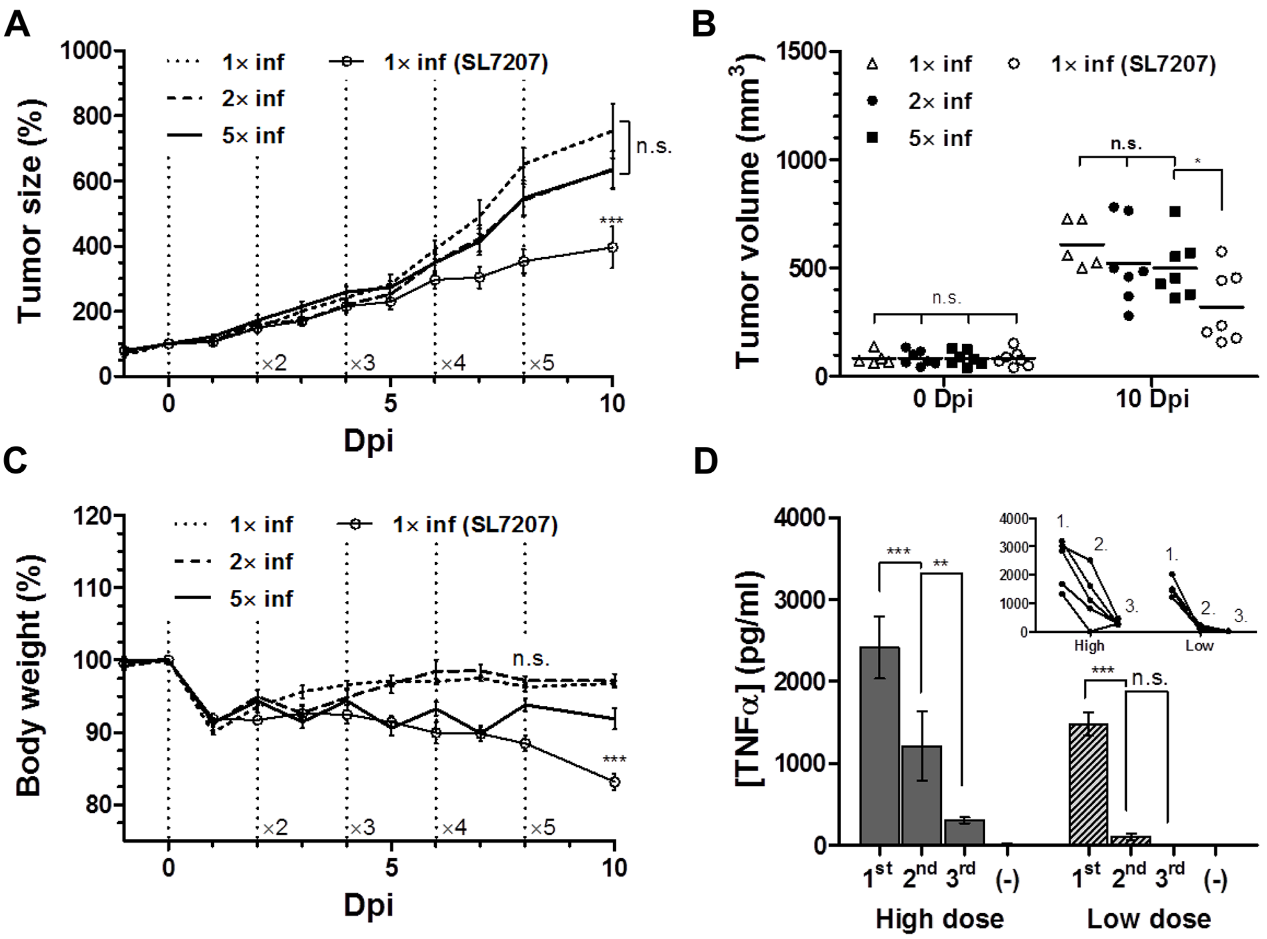

Supplementary Figure S12: Single dose therapy retains maximum efficiency of Symbioflor-2. RenCa tumor bearing mice were infected with Symbioflor-2 representative strain G1/2 through single or multiple inoculation(s). Re-infection was performed with two day intervals, as indicated. **A.** RenCa tumor development over the course of infection with different treatment procedures. Time points of re-infection are indicated with braded vertical lines. **B.** Endpoint tumor volume does not reveal significant difference between single and multiple infections. **C.** Re-infections are transiently reflected in the body weight of the host. **D.** Serum TNFα levels 1.5 h post each sequential infection on day 0, 2, and 4 p.i. as determined using ELISA. High, and low infection dose signify $5x10^7$ and $5x10^6$, respectively. The miniature graph illustrates the dependence between single replicates. (-) indicates control serum isolated at day 2 p.i. Displayed are mean ± SEM. N=5-7.

Supplementary Table S1: Genomic comparison between Symbioflor-2 and EcN

Gene cluster	Description	*E. coli* G1/2	*E. coli* G3/10	*E. coli* G4/9	*E. coli* G5	*E. coli* G6/7	*E. coli* G8	*E. coli* Nissle1917	References
ADHERENCE FACTORS AND BIOFILM FORMATION									
csg (csgABCDEFG)	Curli fimbriae	-[1]	+	-[1]	-[1]	-[1]	-[1]	+	[1, 2]
fim (fimABCDEFGI)	Type 1 fimbriae	-	+	+	+	-	-	+	[3]
mrk (mrkABCDEF)	Type 3 fimbriae	-	+[2]	-	-	-	-	-	[3]
iha	Bifunctional enterobactin receptor/ adhesin	+	-	-	-	+	+[3]	+	[3]
upaG	Trimeric autotoransporter adhesin	-	-	-	-	-	-	+	[4]
IRON UPTAKE SYSTEMS									
iuc (iucABCT, iutA)	Aerobactin	+	-	-	-	+	+	+	[2, 3]
chu (chuASTUVWXY)	*E. coli* hemin uptake system	-	-	-	-	-	-	+	[2]
ent (entABCDEF, fepABCDEG)	Enterobactin	+	+	+	+	+	+	+	[2, 3]
fec (fecABCDEIR)	Ferric dicitrate transport system	+	-	+	+[4]	+	+	+	[2, 3]
iro (iroBCDEN)	Salmochelin	-	-	-	-	-	-	+	[2]
ybt (ybtAEPQSTUX, irp1, irp2, fyuA)	Yersiniabactin	-	-	-	-	-	-	+	[2]
TOXINS									
cib	Colicin-Ib	+	-	-	-	+	+	-	[3]
csa, csi, csl	Colicin S4	+[5]	-	-	-	+[5]	+[5]	-	[3]
mch (mchBCDEFIX)	Microcin H47	-	-	-	-	-	-	+	[5]
mcm (mcmAIM)	Microcin M	-	-	-	-	-	-	+	[5]
mcs (mcsABIS)	Microcin S	-	+[6]	-	-	-	-	-	[6]
hly (hlyABCD)	*E. coli* hemolysin A	+	-	-	-	+	+	-	[3, 7, 8]
MISCELLANEOUS									
eib (eibACDEFG)	Immunoglobulin binding, increased serum survival	-[7]	-	-	-	-[7]	-[7]	-	[9]
gad (gadA, gadBC, gadE, gadXW)	Glutamate decarboxylase	+[8]	+[9]	+[10]	+	+[11]	+	+	[3]

[1] incomplete, missing *csgBC*; [2] encoded on plasmid pSYM1 (accession no. JN887338), missing *mrkE*; [3] truncated *iha*; [4] truncated *fecI*; [5] encoded on plasmid pSYM12 (accession no. KM107848); [6] encoded on plasmid pSYM1; [7] incomplete locus containing truncated *eibACDF* and missing *eibEG*; [8] no *gadA*; [9] truncated *gadX*; [10] no *gadBC*; [11] truncated *gadA*
